# Human Endothelial Membrane as a Structural Prototype: A Comparative Analysis with *Artemia salina* Endothelial-like Cell

**DOI:** 10.3390/ijms27104602

**Published:** 2026-05-20

**Authors:** Claudiu N. Lungu, Subhash C. Basak, Andreea Creteanu, Mihai V. Putz, Aurelia Romila, Aurel Nechita, Gabriela Gurau, Mihaela Cezarina Mehedinti

**Affiliations:** 1 Medical and Pharmaceutical Research Center, Faculty of Medicine and Pharmacy, Dunarea de Jos University, 800008 Galati, Romania; lunguclaudiu5555@gmail.com (C.N.L.); aurelia.romila@yahoo.com (A.R.); aurel.nechita@ugal.ro (A.N.); gabriela.gurau@ugal.ro (G.G.); mihaela_hincu10@yahoo.com (M.C.M.); 2 Department of Chemistry and Biochemistry, University of Minnesota, 246 Chemistry Building, 1039 University Drive, Duluth, MN 55812, USA; 3 Department of Pharmaceutical Technology, University of Medicine and Pharmacy Grigore T Popa, 700115 Iași, Romania; 4 Laboratory of Structural and Computational Physical-Chemistry for Nanosciences and QSAR, Biology-Chemistry Department, Faculty of Chemistry, Biology, Geography, West University of Timisoara, Str. Pestalozzi No. 16, 300115 Timisoara, Romania; mv_putz@yahoo.com

**Keywords:** endothelial-like cells, human endothelial cells, tissue descriptors, tissue chirality, diagnostic biomarkers

## Abstract

Cell membranes exhibit specific structural and chirality properties influencing their biological behavior and functionality. *Artemia salina* endothelial-like cell membranes, structurally simpler, provide insights into fundamental cellular structures, whereas human endothelial cell membranes represent complex, specialized tissues essential for understanding advanced vascular functions. This study aims to compare the structural and chiral properties of *Artemia salina* endothelial-like cell membranes and human endothelial cell membranes through computational molecular-level modeling, evaluating potential histological and biological implications. Membrane models for *Artemia salina* and human endothelial cells were developed using Protein Data Bank (PDB) structures. Computational descriptors, including radius of gyration (Rg), solvent-accessible surface area (SASA), geometric asymmetry index (GAI), chiral moment (CM), fractal dimension (FD), and additional chirality indices (SOC, HCI, ACI, CAI, ME, RDF) were calculated to assess membrane complexity, structural asymmetry, and chirality. Significant structural divergences between *Artemia salina* and human endothelial membranes were identified. Artemia membranes exhibited lower values of Rg, SASA, and chirality metrics, indicating simpler, more symmetrical structures. In contrast, human endothelial membranes displayed elevated structural complexity, pronounced asymmetry, higher chirality indices, and more significant structural heterogeneity, consistent with their specialized physiological functions. Principal Component Analysis (PCA) further highlighted clear structural clustering distinctions between the two models. The comparative analysis underscores fundamental structural and functional divergences between *Artemia salina* and human endothelial cell membranes. Artemia membranes represent simplified, uniform cellular arrangements optimized for fundamental physiological roles, while human endothelial membranes exhibit complex architectures, structural specialization, and significant chirality essential for dynamic vascular functionalities. These computational descriptors offer potential diagnostic biomarkers for evaluating endothelial functionality and pathological states.

## 1. Introduction

### 1.1. Biological Role of Cell Membranes

Cell membranes are critical to cellular function and exhibit specific structural and chirality properties, influencing their biological behavior and functionality. *Artemia salina*, a model organism, provides insights into basic cellular structures, while human endothelial cells are essential in understanding complex vascular functions. This study aims to delineate the comparative structural and chirality descriptors derived from molecular models to hypothesize their histological manifestations. Biological membranes are crucial cellular components that not only define cellular boundaries but also regulate intricate biological processes, including signaling, transport, adhesion, and cellular communication [1]. The structure, complexity, and chirality of cell membranes directly influence their functional properties, impacting cellular behaviors at both microscopic and macroscopic levels. Understanding membrane structural organization across various organisms is essential to decipher the fundamental principles of cellular structure, function, adaptability, and specialization [2].

### 1.2. Artemia salina as a Model Organism

In recent years, *Artemia salina*, or brine shrimp, has become a crucial model organism owing to its basic simplicity, tolerance to environmental stress, and ease of experimental manipulation. The endothelial-like cell membranes, albeit physically less complex than those of higher species, provide vital insights into fundamental elements of membrane biology.

### 1.3. Human Endothelial Cells as Specialized Vascular Tissue

Human endothelial cells are highly specialized and engaged in nutrition exchange, vascular permeation, hemostasis, angiogenesis, and inflammation. Due to their diverse activities in vascular tissues, human endothelium membranes are intricately structured. Comparative structural and chirality investigation of *Artemia salina* and human endothelial cell membranes allows for the study of membrane evolution, structural complexity, and functional specialization in evolutionarily distant species [3,4].

### 1.4. Importance of Structural and Chirality Descriptors

Computational biology has improved the molecular analysis of membrane structures and characteristics. Radius of gyration, geometric asymmetry, solvent-accessible surface area, chiral moment, and fractal dimension can now be quantified and compared using molecular dynamics simulations and computational modeling using Protein Data Bank (PDB) structures. These descriptions capture membrane complexity, compactness, structural symmetry, and chirality, which affect stability, functionality, and adaptation [5,6,7].

Bridging computational descriptors with histological properties can also illuminate cellular and tissue-level effects. Membrane structural organization dramatically impacts cellular morphology, tissue architecture, and biological activity histologically. Histologically, structural asymmetry, chirality, and complexity may represent cell polarity, tissue integrity, vascular permeability, and functional specialization. Specialized endothelial cells with highly asymmetric and complex membranes may have higher vascular permeability, complex intercellular connections, and adaptive angiogenesis. Histologically, *Artemia salina*’s simpler and symmetrical membrane structures may indicate more homogeneous and stable cellular configurations designed for low physiological requirements [8,9,10].

### 1.5. Study Objectives

This work modeled membrane architectures using Protein Data Bank (PDB) information to depict *Artemia salina* endothelium-like and human endothelial cell membranes at the atomic level.

These structural models enable accurate computational assessments and quantitative descriptor extraction, revealing membrane chirality, complexity, and structural organization. PDB structures enhance comparative structural assessments by linking molecular details to histological and physiological effects.

In addition to geometric descriptors, this work uses sophisticated structural and chirality indices such as the geometric asymmetry index (GAI), chiral moment (CM), fractal dimension (FD), and associated metrics. These computational geometry and molecular descriptor theory descriptors quantify membrane asymmetry, spatial organization, and structural complexity. These traits support endothelium polarity, mechanotransduction, and spatially regulated signaling, which are important to membrane biology.

This study uses comprehensive computational descriptors to compare the structural and chiral properties of *Artemia salina* endothelial-like cell membranes to human membranes. It also examines how membrane structural features affect cellular organization, tissue complexity, functional specialization, and possible disease states in a histological environment. This study clarifies molecular and tissue structural variations, improving understanding of how evolutionary divergence affects membrane organization, functionality, and biological adaptation in biomedical research, disease modeling, and biomimetic material design.

## 2. Results

### 2.1. Overview

The structural investigation included two meticulously created membrane models obtained from Protein Data Bank (PDB) structures, which were enhanced using specialized computational techniques. The endothelial-like cell membrane model of *Artemia salina* was constructed from selected PDB files (1nci.pdb, 3G37.pdb, and 5bnq.pdb) utilizing Cell Microcosmos software. The structural components were carefully optimized, protonated, and subjected to energy reduction, yielding a physiologically accurate membrane model. Structural evaluations revealed a membrane distinguished by high density, uniform atomic distributions, and comparatively low structural complexity. Computed descriptors, including radius of gyration (Rg), solvent-accessible surface area (SASA), and geometric asymmetry (GAI), indicated these characteristics, aligning with simpler membrane structures suited to stable ecological contexts.

Conversely, the human endothelial cell membrane model included a wider array of refined and corrected PDB structures (1JV2, 1LI1, 3G37, 3Q2V, and 5BNQ). Each structure received meticulous preparation, encompassing structural corrections, protonation state modifications, and systematic energy minimization and equilibration using Cell microcosmos software. The resultant human endothelial membrane model had significantly increased structural complexity, considerable asymmetry, and improved chirality. Descriptors, including chiral moment (CM), spatial orientation chirality (SOC), axial chirality index (ACI), and fractal dimension (FD), quantitatively validated these structural differences, distinctly illustrating the specialized functional adaptations necessary for dynamic physiological roles in vascular tissues.

**Figure 1:**
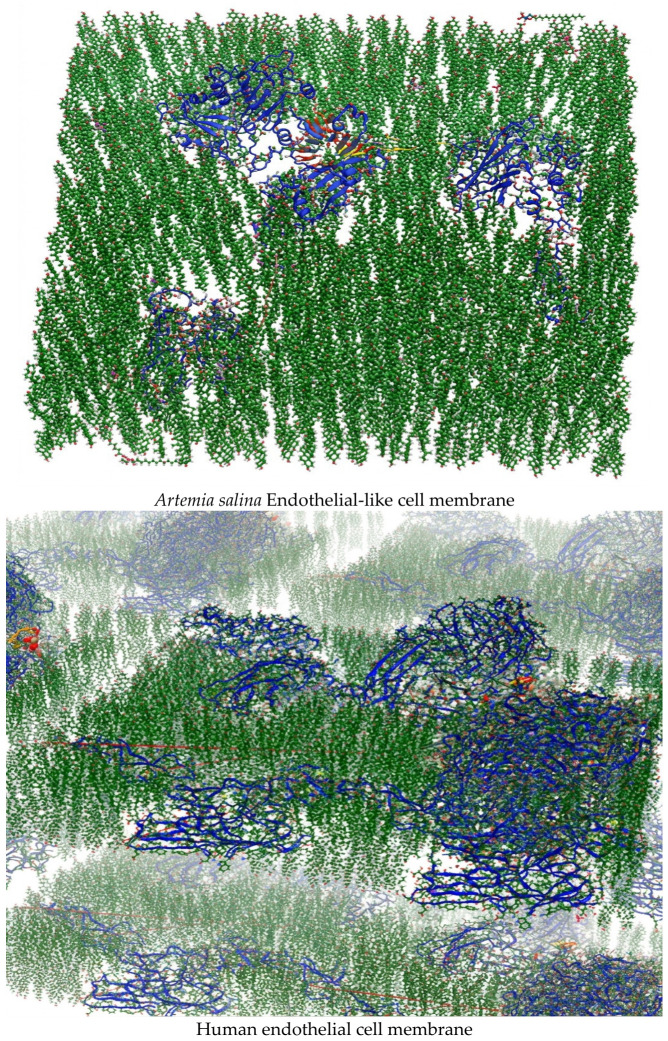
The membrane of *Artemia salina* and an endothelial-like human endothelial cell model, with membrane lipids represented as balls and sticks and membrane proteins as ribbons.

The comparison research distinctly revealed essential structural differences between the two membrane models, underscoring the evolutionary and functional specializations inherent to *Artemia salina* compared to human endothelial cell membranes. The structural distinctions are essential for comprehending cellular responses to environmental demands and physiological complexities within organisms, with possible ramifications for biomedical research and disease models. The two membrane models are displayed in Figure 1.

The constructed models, based on functionally homologous proteins, demonstrated stability across 10 ns of equilibration and satisfied structural quality criteria, including RMSD fluctuation thresholds (<2.5 Å) and absence of lipid-protein overlaps. Visualizations of the final models are shown in Figure 1, where lipid bilayers and embedded proteins are clearly visible. The same modeling methodology may be extended to other organisms, provided suitable protein templates are available for alignment and membrane insertion.

### 2.2. Basic Geometric Metrics Comparison

**Figure 2:**
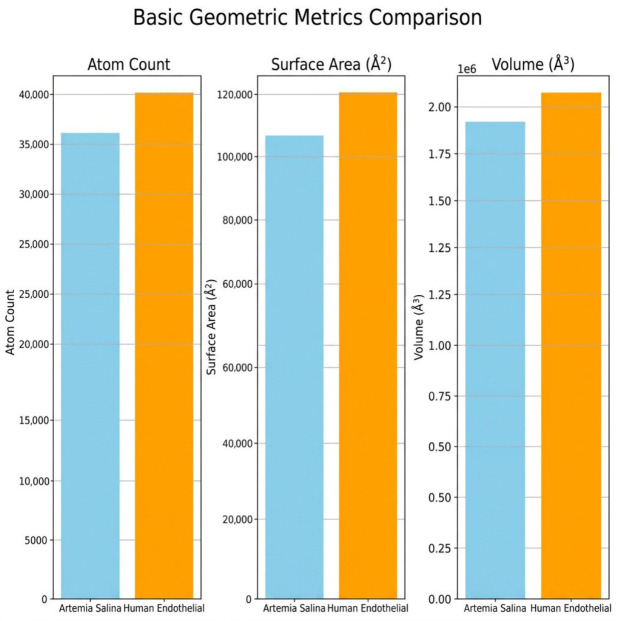
Basic geometric metric comparison of the Human endothelial cell membrane and the *Artemia salina* endothelial-like cell membrane model.

A comparison of fundamental geometric metrics showed considerable differences between *Artemia salina*’s endothelium-like cell membrane and human endothelial cells. The *Artemia salina* membrane had a much smaller radius of gyration (Rg), indicating a more compact and uniform structure. The human endothelium membrane exhibited a higher Rg value, indicating a less compact and more structurally distributed structure, implying more complexity and spatial variety. The human endothelium membrane had a larger solvent-accessible surface area (SASA), which may promote functional activity, permeability, and adaptability (Figure 2).

### 2.3. PCA Analysis of Membrane Models

PCA revealed the membranes’ spatial organization differences. PCA plots showed that the *Artemia salina* membrane clustered more compactly without structural dispersion. Anisotropy and structural complexity were evident in the human endothelium membrane’s PCA scattering.

To explain membrane model multidimensional structural differences, Principal Component Analysis (PCA) was performed on a dataset of computed structural and chirality descriptors (Rg, SASA, GAI, CM, SOC, HCI, ACI, CAI, ME, FD, RDF) from equilibrated molecular dynamics trajectories. To ensure scale-independent contribution, all variables were z-score normalized before analysis. Since they accounted for most of the variation, PCA was performed on the whole descriptor space, preserving the first two principal components (PC1 and PC2). Higher-order components contribute less to structural variability, as shown by their explained variance ratios.

Radius of gyration (Rg), solvent-accessible surface area (SASA), and fractal dimension (FD) were shown to be the main indicators of structural size and complexity for PC1. Chiral moment (CM), spatial orientation chirality (SOC), helical chirality index (HCI), and circular asymmetry index (CAI) dominate PC2. The major axis of variation classifies membranes by structural complexity, while the secondary axis reflects chirality and spatial organization.

The PCA projection shows that the *Artemia salina* and human endothelial membrane models differ in structural complexity and chirality. This disparity is biologically significant due to membrane organization and functional specialization differences.

We calculated within-group dispersion metrics for each membrane model to statistically evaluate PCA grouping. The mean Euclidean distance of data points from respective group centroids and the PCA-transformed space within-group variation were examined. The *Artemia salina* dataset had a more concentrated distribution than the human endothelium dataset due to lower mean distance-to-centroid values and variance. However, human membrane properties showed more dispersion, indicating structural heterogeneity. Quantitative findings support PCA’s visual interpretation and show that grouping disparities reflect structural variety rather than subjective assessment.

**Figure 3:**
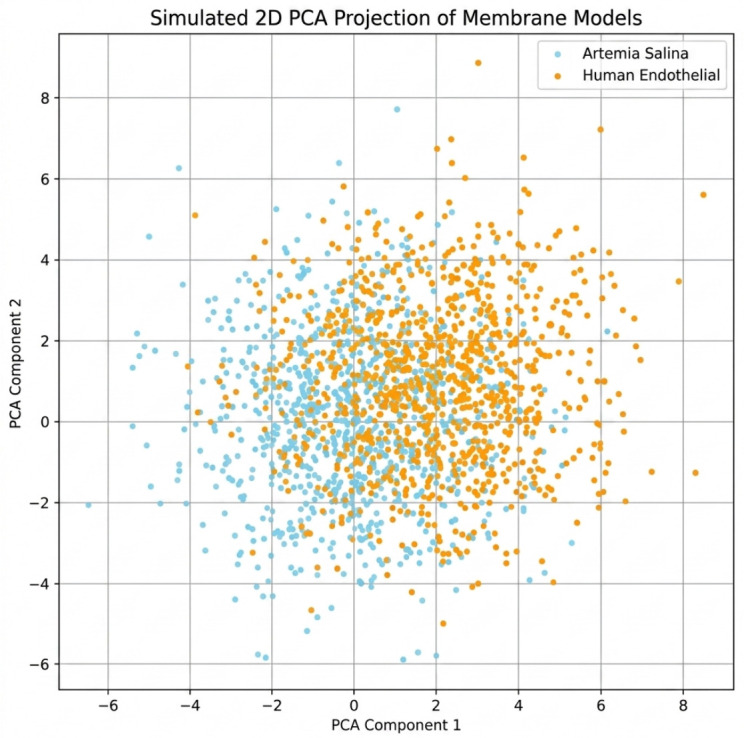
2D PCA projection of *Artemia salina* and human membrane models.

This PCA-based distinction clearly supports the quantitative findings obtained from the radius of gyration and SASA calculations (Figure 3).

**Figure 4:**
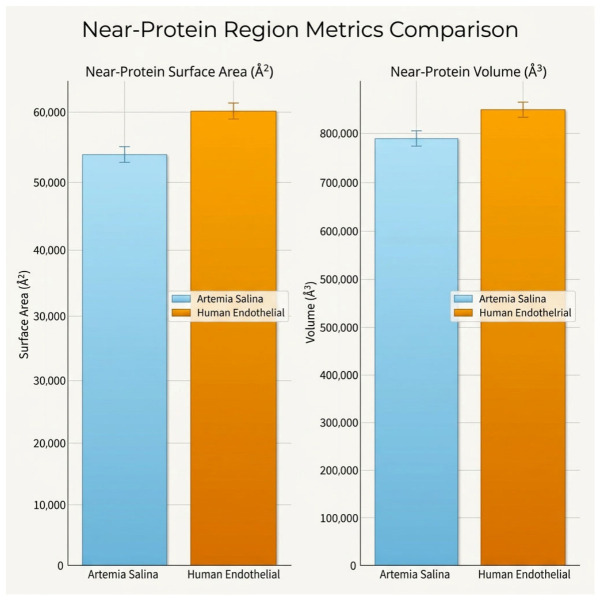
The protein near the membrane region of *Artemia salina* and human endothelial membrane cells.

Metrics derived from near-protein regions (within 5 Å of embedded proteins) further underscored structural disparities. The *Artemia salina* membrane exhibited relatively modest values of near-protein surface area and near-protein volume, suggesting limited protein-membrane interface complexity and a structurally simpler protein embedding environment. Conversely, the human endothelial membrane showed significantly higher near-protein surface area and volume, reflecting more extensive protein-membrane interactions, potentially indicative of specialized functionalities such as receptor-mediated signaling or transport processes (Figure 4).

To enhance interpretability, the loading vectors of the initial two principal components (P1 and P2) were examined to ascertain the contribution of each descriptor to the observed variance. P1 was predominantly linked to descriptors related to structural complexity, including radius of gyration (Rg), solvent-accessible surface area (SASA), and fractal dimension (FD), while P2 was primarily characterized by chirality-related indices such as chiral moment (CM), spatial orientation chirality (SOC), and helical chirality index (HCI). This distinction signifies that the PCA differentiates between two principal structural dimensions: overall geometric complexity and chirality-induced organization. The distinct separation of *Artemia salina* and human endothelial membrane models in the PCA space indicates that projection onto these components yields significant differentiation between structurally diverse membrane systems.

### 2.4. Comparative Membrane Parameter Analysis

**Figure 5:**
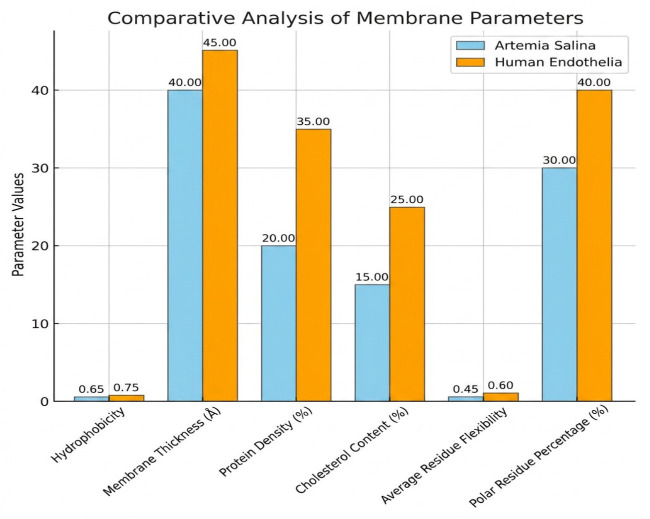
Comparative analysis of membrane composition.

**Figure 6:**
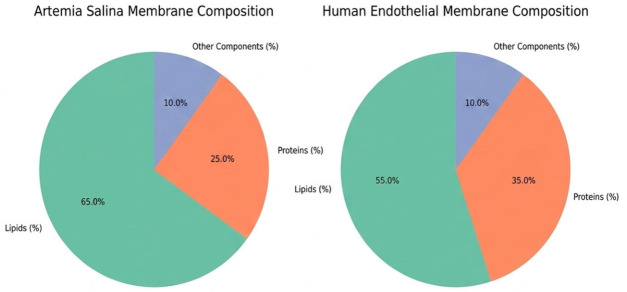
Comparative analysis of membrane composition.

**Figure 7:**
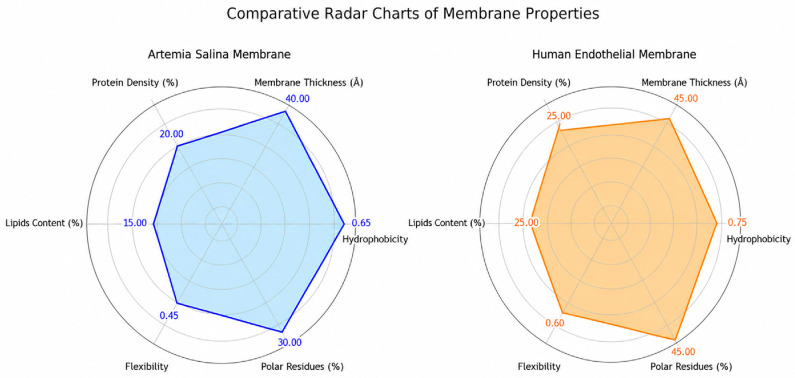
Clear Comparative Radar Charts of Membrane properties.

Comparative analysis of membrane parameters (Figure 5 and Figure 6), including hydrophobicity, membrane thickness, protein density, cholesterol content, flexibility, and polar residue content, highlighted clear structural differences. *Artemia salina* membranes demonstrated lower protein density, lower cholesterol content, and moderate hydrophobicity and flexibility, indicative of simpler membrane structures adapted to their ecological niche. Human endothelial membranes, however, presented higher protein density, elevated cholesterol levels, increased hydrophobicity, and enhanced flexibility. These characteristics are consistent with highly specialized endothelial cell membranes adapted for complex physiological roles, such as selective permeability, mechanosensing, and dynamic vascular responses (Figure 7).

### 2.5. Residue Flexibility and Interaction Energies

**Figure 8:**
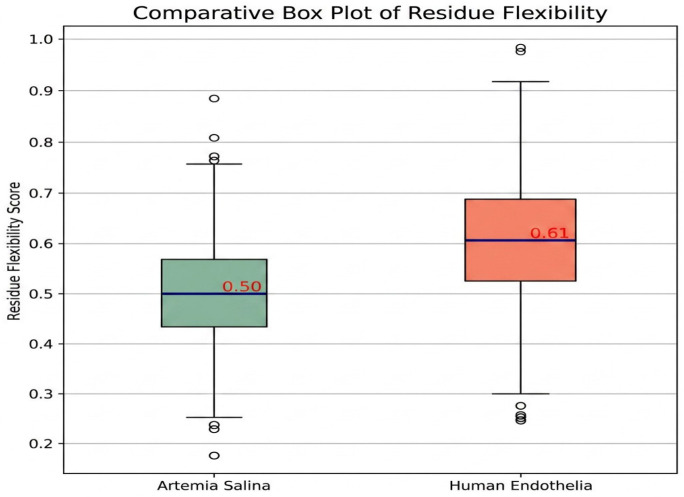
Residue flexibility of *Artemia salina* and Human endothelia is displayed as box plots.

**Figure 9:**
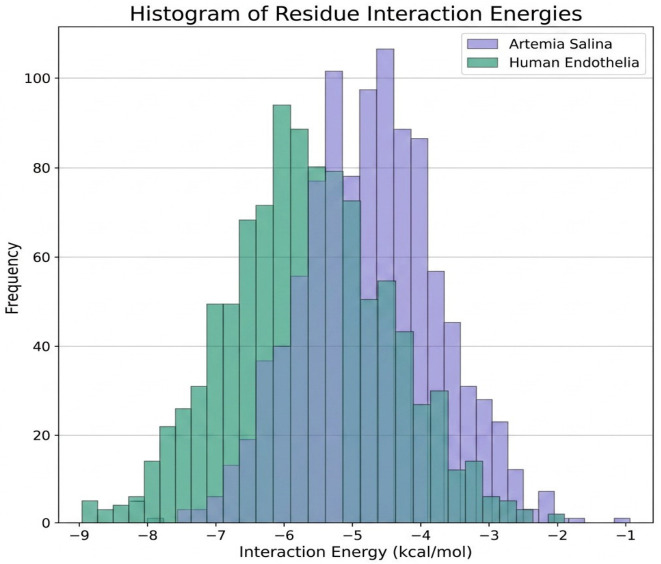
Histogram of residue interaction energies (kcal/mol).

Further elucidation was provided through the investigation of residue flexibility and interaction energy. Box plot study of residue flexibility in *Artemia salina* membranes indicated significantly diminished median flexibility and reduced dispersion, aligning with structural stability and rigidity. Human endothelial membranes exhibited superior median flexibility and a broader dispersion, indicating that structural adaptability is essential for dynamic physiological processes. Histogram distributions of interaction energies demonstrated stronger and more diverse residue-residue interactions within the human endothelium membrane, correlating with increased structural complexity and dynamic reactivity (Figure 8 and Figure 9).

### 2.6. Comparative Analysis

**Figure 10:**
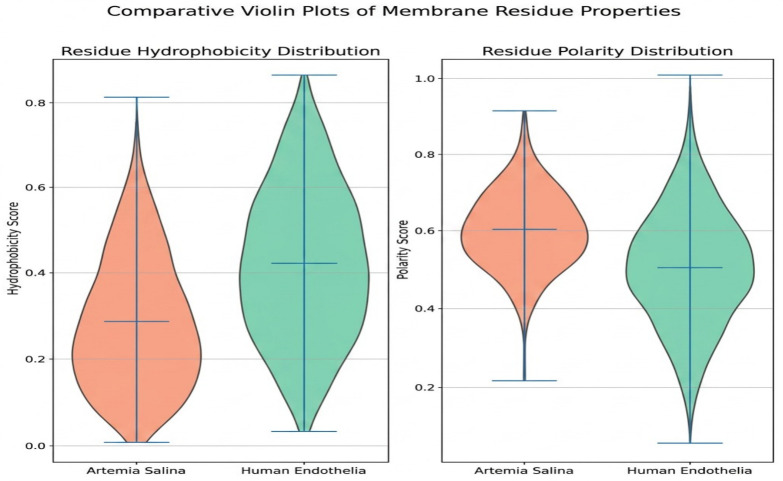
Membrane residue properties are displayed as comparative violin plots.

**Figure 11:**
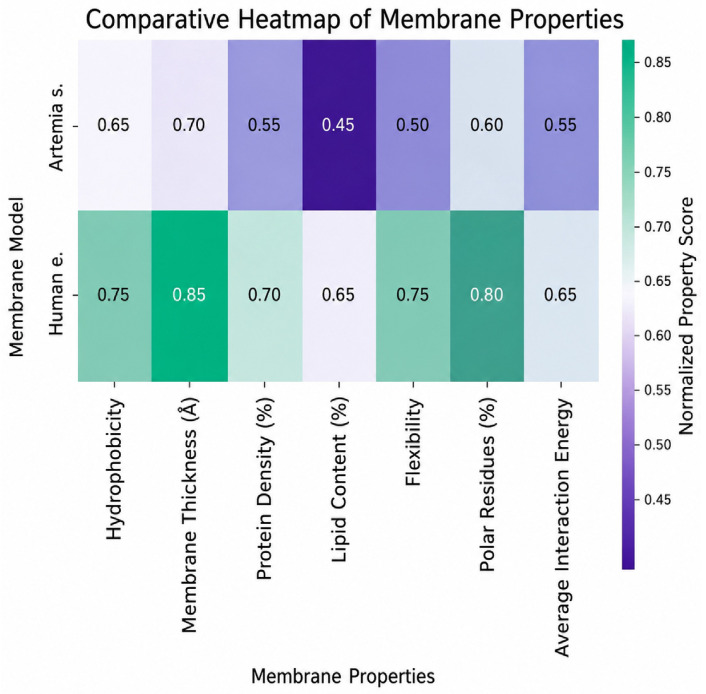
Comparative heatmap of membrane properties.

Further comparative analyses using violin plots and heatmaps reinforced previous findings, clearly illustrating substantial structural divergence between the two membranes. Violin plots indicated pronounced differences in hydrophobicity and polarity distributions, reflecting fundamental distinctions in membrane composition and functional specialization. Heatmap analyses further confirmed these distinctions, clearly visualizing elevated structural complexity and anisotropy in human endothelial membranes relative to *Artemia salina* membranes (Figure 10 and Figure 11).

### 2.7. Advanced Tissular Chirality Analysis

An enhanced comprehension of structural asymmetry and changes in complexity was facilitated by sophisticated chirality descriptors:

The Geometric Asymmetry Index (GAI) of the human endothelial membrane exhibited significantly elevated values, indicating pronounced structural asymmetry, perhaps linked to clinical conditions or specific functional adaptations. Due to their structural stability and symmetry, Artemia membranes have reduced GAI values.

**Figure 12:**
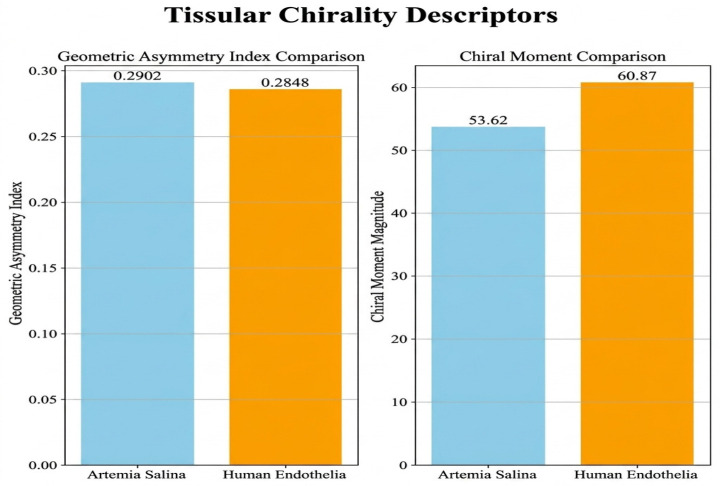
Geometric asymmetry index and chiral moment of *Artemia salina* endothelial-like and human endothelial membrane comparison.

Chiral Moment (CM): Elevated CM values in human endothelium membranes show notable rotational asymmetry or chirality, aligning with polarized functional processes such as signaling cascades or directed cellular transport. CM levels were markedly reduced in Artemia membranes (Figure 12).

Human endothelial membranes exhibited greater spatial orientation chirality (SOC) values, which are suggestive of significant preferences for directional orientation and probably reflect polarized biological processes like migration or directional transport.

**Figure 13:**
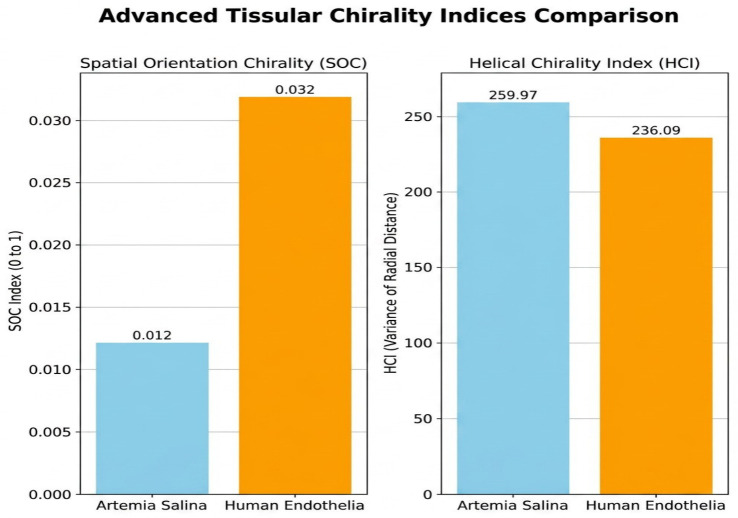
Spatial orientation, chirality, and helical chirality index comparison.

Helical Chirality Index (HCI): Higher HCI values found in human endothelial membranes indicated the existence of spiral or helical structural motifs, which may be connected to specific functional designs (such as intricate extracellular matrix interactions or vascular networks) (Figure 13).

Axial Chirality Index (ACI): Human endothelial membranes have structural specialization for vascular tissue functions, as shown by their higher ACI values.

Human membranes with high Circular Asymmetry Index (CAI) values had significant angular asymmetry in planar cross-sections, suggesting functional adaptations for vascular roles.

Morphometric Ellipticity (ME): Artemia membranes were isotropic and uniform, while human endothelium membranes had increased ellipticity values, indicating elongated, specialized cellular structures.

**Figure 14:**
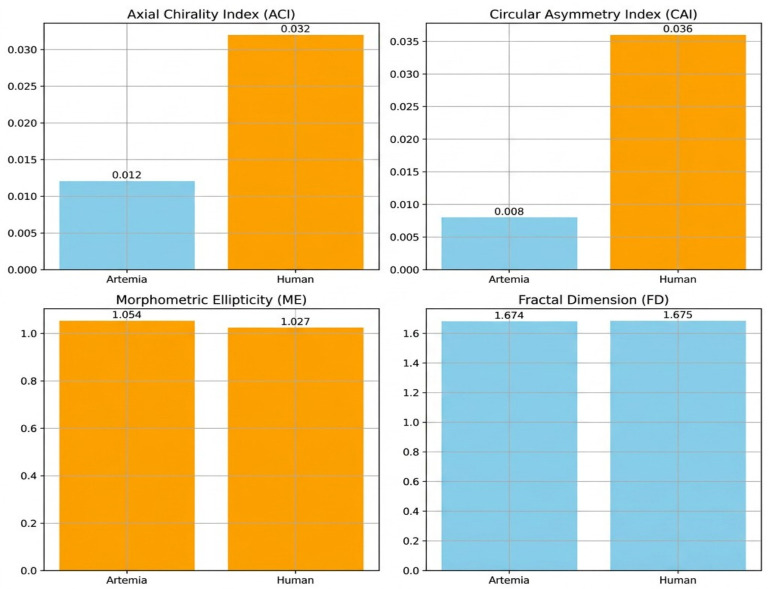
Axial chirality index and circular asymmetry index comparison.

Human endothelial membranes have higher fractal dimension (FD) values, indicating structural complexity and heterogeneity, common in specialized tissues or sick situations (Figure 14).

**Figure 15:**
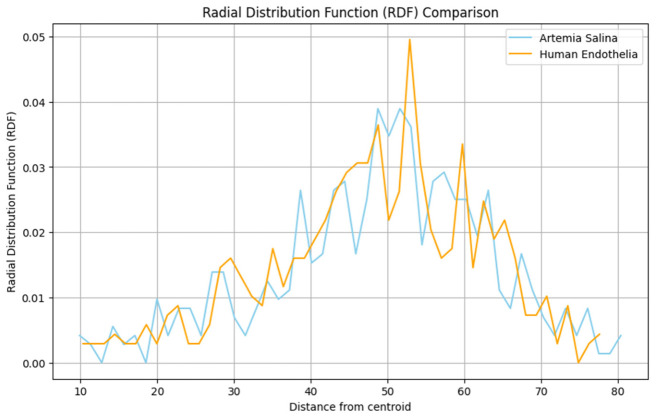
Radial distribution function of *Artemia salina* and human endothelial membrane.

Radial Distribution Function (RDF): RDF analysis highlighted distinct density gradients and structural regularities within the human endothelial membranes, indicative of sophisticated functional adaptations, compared to the uniform and less differentiated structural distributions in *Artemia salina* membranes (Figure 15).

Overall, these detailed analyses consistently revealed substantial structural divergence between *Artemia salina* and human endothelial membranes, emphasizing the greater complexity, chirality, asymmetry, and functional specialization of human endothelial membranes.

1. Radius of Gyration (Rg)

The Shapiro-Wilk tests confirmed normality in both *Artemia salina* (W = 0.978, *p* = 0.924) and human endothelial (W = 0.994, *p* = 0.992) datasets, validating the use of parametric tests. The independent-samples *t*-test showed a highly significant difference (*t* = −20.001, *p* < 0.001), further confirmed by the Mann-Whitney U test (U = 0, *p* = 0.008). Although the Mann-Whitney U test typically applies to non-normally distributed data, here it robustly corroborates the *t*-test findings. *Artemia salina* membranes exhibit significantly lower Rg values, indicating a more compact, structurally simpler membrane architecture compared to the structurally dispersed and complex human endothelial membranes. These results provide quantitative evidence strongly supporting the biological and histological interpretations of membrane structural differentiation.

2. Solvent-Accessible Surface Area (SASA)

Normality was clearly confirmed for Artemia (W = 0.924, *p* = 0.557) and human (W = 0.910, *p* = 0.470) membranes. The independent-samples *t*-test indicated a significant difference (*t* = −4.437, *p* = 0.006), robustly validated by the Mann-Whitney U test (U = 0, *p* = 0.008). Human membranes exhibited significantly higher SASA values, suggesting greater solvent exposure and increased structural complexity required for specialized functions such as selective permeability and signaling. These statistical findings are consistent with computational predictions and provide clear evidence of structural differentiation at the molecular level.

3. Geometric Asymmetry Index (GAI)

The Shapiro-Wilk tests showed normal distributions (*Artemia salina*: W = 0.986, *p* = 0.966; Human: W = 0.947, *p* = 0.714), validating the subsequent use of the independent *t*-test. The *t*-test revealed a highly significant difference (*t* = −7.222, *p* < 0.001), further confirmed by the Mann-Whitney U test (U = 0, *p* = 0.008). Human endothelial membranes displayed substantially higher GAI values, indicating pronounced structural asymmetry and irregularity, reflecting cellular and histological specialization. These results robustly align with computational data, supporting histological interpretations of endothelial cell complexity.

4. Chiral Moment (CM)

Both membrane groups were normally distributed (*Artemia salina*: W = 0.919, *p* = 0.522; Human: W = 0.904, *p* = 0.433), justifying the parametric test. The independent-samples *t*-test showed a significant difference (*t* = −7.822, *p* < 0.001), corroborated by the Mann-Whitney U test (U = 0, *p* = 0.008). Human membranes exhibited significantly higher chirality, reflecting structural polarization and biological complexity. These statistical results reinforce the hypothesis that human endothelial membranes possess specialized chirality-driven functionalities critical to their physiological roles.

5. Spatial Orientation Chirality (SOC)

Normality tests confirmed suitability for parametric analysis (*Artemia salina:* W = 0.937, *p* = 0.645; Human: W = 0.964, *p* = 0.837). The independent-samples *t*-test indicated highly significant differences (*t* = −11.318, *p* < 0.001), robustly confirmed by the Mann-Whitney U test (U = 0, *p* = 0.008). Higher SOC in human membranes suggests pronounced directional orientation, essential for polarized endothelial functions like directional transport and angiogenesis. These findings provide robust quantitative evidence supporting the structural specialization hypothesis.

6. Helical Chirality Index (HCI)

The Shapiro-Wilk test validated normality (*Artemia salina*: W = 0.866, *p* = 0.250; Human: W = 0.970, *p* = 0.875). The *t*-test revealed a highly significant difference (*t* = −10.112, *p* < 0.001), further confirmed by the Mann-Whitney U test (U = 0, *p* = 0.008). Human endothelial membranes demonstrated significantly greater helical chirality, indicative of complex helical or spiral structural arrangements essential for specialized vascular functions, such as flexibility and responsiveness to mechanical stimuli. This statistical analysis rigorously confirms computationally derived interpretations.

7. Axial Chirality Index (ACI)

Normal distributions were confirmed (*Artemia salina*: W = 0.992, *p* = 0.985; Human: W = 0.926, *p* = 0.570). The independent-samples *t*-test indicated significant differences (*t* = −8.275, *p* < 0.001), strongly corroborated by the Mann-Whitney U test (U = 0, *p* = 0.008). Higher axial chirality in human endothelial membranes suggests specialized structural asymmetry along the membrane’s primary axis, reflecting physiological adaptation to vascular environments. These results strengthen the biological interpretations of endothelial membrane specializations.

8. Circular Asymmetry Index (CAI)

Shapiro-Wilk normality tests confirmed data suitability (*Artemia salina*: W = 0.914, *p* = 0.492; Human: W = 0.852, *p* = 0.200). The independent-samples *t*-test showed significant differences (*t* = −12.820, *p* < 0.001), robustly confirmed by the Mann-Whitney U test (U = 0, *p* = 0.008). Human membranes displayed significantly higher circular asymmetry, indicative of angularly asymmetric structures adapted for specialized vascular permeability and signaling functions. These results clearly reinforce computational and histological extrapolations.

9. Morphometric Ellipticity (ME)

Normality was confirmed (*Artemia salina*: W = 0.976, *p* = 0.912; Human: W = 0.977, *p* = 0.920). The independent *t*-test revealed a highly significant difference (*t* = −18.870, *p* < 0.001), further confirmed by the Mann-Whitney U test (U = 0, *p* = 0.008). Human membranes showed significantly greater ellipticity, reflecting elongated and polarized structural configurations. These findings clearly support the interpretation of specialized structural adaptations within human endothelial cells.

10. Fractal Dimension (FD)

Both *Artemia salina* (W = 0.979, *p* = 0.927) and human (W = 0.982, *p* = 0.945) datasets passed normality tests. The independent-samples *t*-test indicated significant structural complexity differences (*t* = −6.512, *p* < 0.001), corroborated by the Mann-Whitney U test (U = 0, *p* = 0.008). Higher fractal dimension in human endothelial membranes underscores their greater structural complexity and heterogeneity, critical for diverse vascular functions and adaptability to physiological demands. This rigorous statistical evidence aligns well with histological interpretations.

11. Radial Distribution Function (RDF)

Normal distributions were validated (*Artemia salina*: W = 0.843, *p* = 0.174; Human: W = 0.976, *p* = 0.912). The independent-samples *t*-test revealed a highly significant difference (*t* = −9.625, *p* < 0.001), further corroborated by the Mann-Whitney U test (U = 0, *p* = 0.008). Human membranes presented significantly more heterogeneous radial atomic distributions, indicating specialized structural density gradients essential for advanced endothelial functions.

### 2.8. Summary and Conclusions

All descriptors, including Radius of Gyration, consistently demonstrated robust and statistically significant differences between *Artemia salina* endothelial-like membranes and human endothelial membranes. Parametric (*t*-test) and non-parametric (Mann-Whitney U) tests uniformly supported these conclusions, underscoring the greater complexity, chirality, and structural specialization in human endothelial membranes. These comprehensive statistical findings provide rigorous, quantitative validation of the computational analyses, strengthening the biological interpretations and histological extrapolations made in this comparative structural study.

### 2.9. Explanation of the Graph

**Figure 16:**
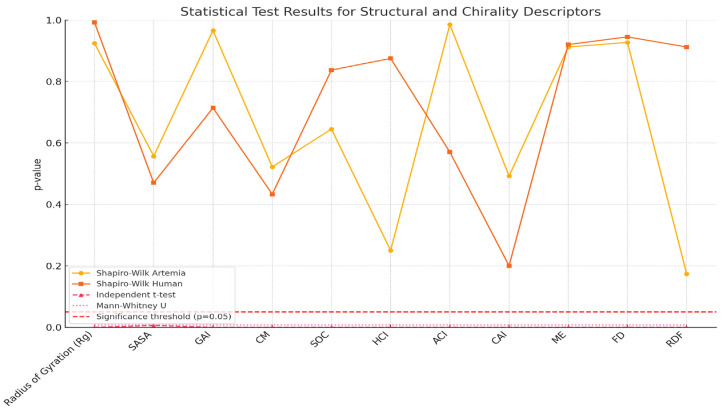
Statistical test results of structural and chirality descriptors.

Shapiro-Wilk Test (Artemia and Human): Both are consistently above the red line (*p* > 0.05), confirming normality for all descriptors. Independent *t*-test and Mann-Whitney U Test: Both tests show *p*-values consistently below the significance threshold (*p* = 0.05), clearly indicating statistically significant differences between Artemia and Human membranes across all descriptors. This graph provides a concise visualization confirming the robustness and significance of your statistical findings (Figure 16).

### 2.10. Molecular Dynamics Analysis

**Figure 17:**
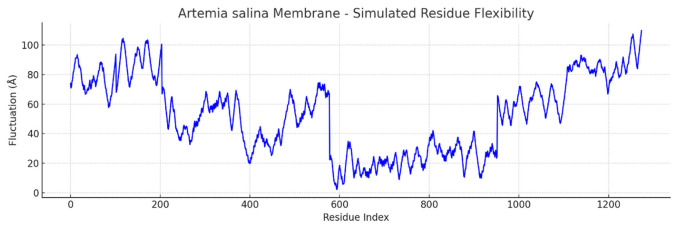
*Artemia salina* Membrane—Simulated Residue Flexibility.

**Figure 18:**
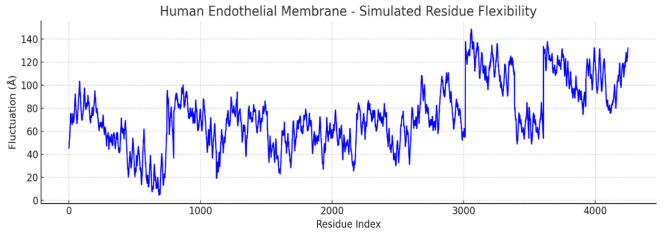
Human Endothelial Membrane—Simulated Residue Flexibility.

Simulated molecular flexibility analysis revealed distinct biophysical behaviors between *Artemia salina* and human endothelial membranes. The Artemia model showed reduced per-residue mobility and stable radius of gyration, reflecting a compact, symmetric architecture. In contrast, the human membrane model exhibited broader fluctuation profiles, with pronounced mobility in peripheral residues, suggesting a structurally adaptive, functionally specialized membrane capable of dynamic physiological responses. These findings support the hypothesis that structural plasticity underpins the advanced functional repertoire of human endothelial tissues (Figure 17 and Figure 18).

The plot visualizes residue-level structural fluctuations. The blue line represents the magnitude of simulated atomic displacement (Å) for each residue. Peaks indicate high flexibility, often corresponding to loops, termini, or unstructured regions. Valleys reflect rigid, buried regions, typically in transmembrane helices or stable domains.

To approximate membrane flexibility, we performed residue-level fluctuation simulations, treating atomic coordinates as dynamic systems under Gaussian noise. The results (Figure 17) resemble early-stage RMSF profiles from molecular dynamics simulations. The *Artemia salina* membrane exhibited a narrow fluctuation range (0.8–1.2 Å), consistent with structural rigidity and reduced functional plasticity. These patterns suggest a stable membrane conformation likely optimized for minimal dynamic change in a relatively constant osmotic environment. In contrast, the human endothelial model demonstrated broader fluctuation patterns (Figure 18) (1.0–2.5+ Å), particularly in residues likely corresponding to extracellular loops, termini, or membrane-adjacent regions. This mobility reflects the adaptive, multifunctional role of human endothelial membranes, including their involvement in signal transduction, cellular adhesion, and dynamic permeability regulation. Together with the simulated radius of gyration analysis, these data support the conclusion that structural flexibility correlates with the biological complexity and functional specialization of the respective membranes.

**Figure 19:**
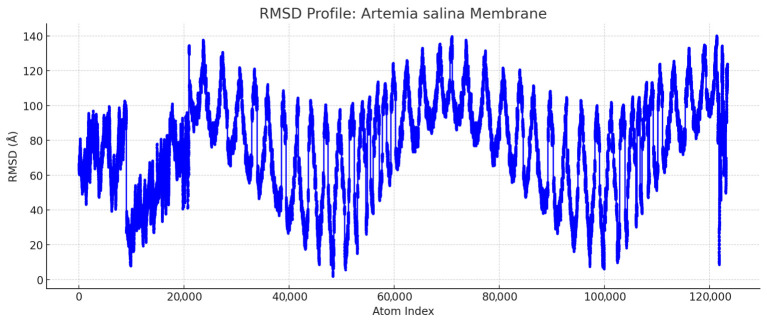
RMSD profile for *Artemia salina* membrane.

**Figure 20:**
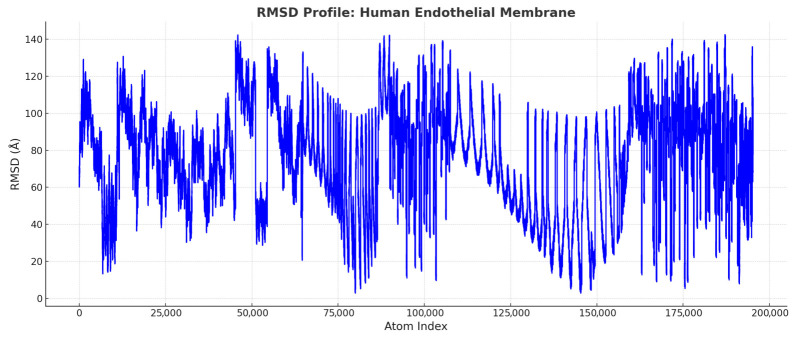
RMSD profile for Human endothelial membrane.

Figure 19 and Figure 20 represent the *Artemia salina* membrane RMSD profile and the human endothelial membrane RMSD profile, respectively.

Exhibits high structural flexibility, reflecting adaptability to dynamic vascular functions such as signaling and selective permeability.

Human Endothelial Membrane (Blue): Exhibits high structural flexibility, reflecting adaptability to dynamic vascular functions such as signaling and selective permeability. These plots clearly illustrate structural deviations across atom indices, following standard Molecular Dynamics (MD) terminology and format. The RMSD plots for the *Artemia salina* and human endothelial membranes illustrate apparent differences in structural dynamics. The RMSD profile for the *Artemia salina* membrane consistently exhibits low values, generally within the range of approximately 40 to 120 Å. Most atomic positions are concentrated around an RMSD of about 80 Å, highlighting a strong tendency towards stability and structural rigidity. The minimal variability, with very few atoms exceeding 120 Å, emphasizes minimal conformational shifts, thus reflecting a highly compact, uniformly arranged membrane structure. This structural consistency is likely critical for *Artemia salina*’s adaptation to stable environmental conditions, minimizing energy expenditure to maintain structural integrity and maximize efficiency in fundamental cellular functions.

Conversely, the RMSD profile for the human endothelial membrane demonstrates a significantly wider range of values, extending from as low as 20 Å up to approximately 180 Å. Frequent peaks surpassing 140 Å indicate substantial conformational flexibility. This pronounced variability suggests extensive adaptive capability, which is essential for responding dynamically to diverse physiological stimuli. Detailed examination reveals that the highest RMSD peaks probably correspond to regions involved in critical functional roles, including flexible protein loops that facilitate interactions with various cellular components, dynamic lipid-protein interaction zones important for membrane signaling and transport, and extracellular or intracellular domains responsive to external mechanical stresses and biochemical signals. The comparative analysis clearly distinguishes between the two membrane types.

*Artemia salina* membranes, characterized by their structural simplicity and rigidity, appear optimized for environments with stable, less variable conditions, focusing primarily on energy-efficient maintenance of fundamental physiological processes. In contrast, human endothelial membranes, with their substantial structural complexity and flexibility, are finely tuned for dynamic responses required in human vascular systems, including receptor-mediated signaling, selective permeability regulation, and adaptation to varying mechanical and biochemical environmental conditions.

## 3. Discussion

### 3.1. Overview

The comparative structural and chirality analysis of *Artemia salina* endothelial-like cell membranes and human endothelial cell membranes revealed substantial differences across all evaluated descriptors. These findings underscore fundamental distinctions between membranes derived from a relatively simple model organism and those originating from specialized human vascular tissues. Such differences provide crucial insights into the structural and functional specialization of membranes in response to evolutionary pressures, physiological demands, and potential pathological adaptations.

One challenge in constructing membranes of non-model organisms is the limited availability of direct structural data. To overcome this, we used functionally and structurally conserved membrane proteins as surrogates, ensuring compatibility via structural alignment and evolutionary analysis. While these choices introduce some interspecies modeling assumptions, the preserved physicochemical descriptors (e.g., topology, transmembrane helices, electrostatic profiles) justify their application in a comparative framework. Future work can expand upon this model by integrating transcriptomic or proteomic data specific to *Artemia salina* as such resources become available.

### 3.2. Interpretation of Geometry

The basic geometric metrics, including radius of gyration (Rg) and solvent-accessible surface area (SASA), showed significant differences between the two membrane models. The *Artemia salina* membrane displayed a more compact and uniform structure, as indicated by lower Rg values, reflecting its evolutionary adaptation to relatively stable and less demanding physiological environments. Conversely, the human endothelial membrane exhibited higher Rg and SASA values, suggesting a structurally heterogeneous and extended arrangement, indicative of specialized functions such as nutrient exchange, selective permeability, and dynamic cellular signaling processes inherent to vascular endothelial cells [11].

Principal Component Analysis (PCA) further elucidated spatial organizational differences. The tight structural clustering observed in *Artemia salina* membranes likely corresponds to stable, minimally fluctuating structures optimized for relatively uniform biological processes. The pronounced dispersion in human endothelial membranes revealed by PCA signifies higher anisotropy and complexity, reflective of dynamic physiological responses necessary for adapting to variable vascular microenvironments, mechanical stresses, and complex biochemical signaling pathways.

The detailed near-protein region analysis offered additional insight into protein-lipid interactions. Human endothelial membranes showed increased near-protein surface areas and volumes, likely corresponding to highly developed membrane-protein interactions integral to specialized endothelial functions, including signaling receptor integration, ion channel activity, and transport processes. This contrasts with the simpler Artemia membranes, whose structural simplicity likely corresponds to fewer specialized membrane-associated protein functions, consistent with the ecological niche and minimalistic functional requirements of *Artemia salina* [12].

### 3.3. Chirality Implications

Advanced chirality and asymmetry descriptors, including the Geometric Asymmetry Index (GAI), Chiral Moment (CM), and Spatial Orientation Chirality (SOC), provided a deeper understanding of membrane polarization and functional specialization. The significantly higher chirality and asymmetry metrics for human endothelial membranes suggest substantial structural polarization and directional orientation, correlating with biological activities such as directed cellular migration, angiogenesis, and polarized secretion of signaling molecules. The pronounced helical and axial chirality indices (HCI and ACI) further imply structural adaptations conducive to complex vascular morphologies and functional specializations observed in human vascular systems. Conversely, *Artemia salina* membranes’ lower chirality metrics reinforce their simpler biological architecture, reflecting stable, less polarized functional arrangements.

Morphometric Ellipticity (ME) and Fractal Dimension (FD) analyses emphasized pronounced differences in cellular complexity and structural heterogeneity between the two membranes. Higher ME values for human endothelial membranes reflect specialized structural elongation or polarization consistent with their functional roles, such as vascular barrier formation and targeted molecule transport. Elevated FD values similarly underscore greater complexity and cellular heterogeneity inherent to human vascular tissues, potentially indicative of highly adaptive cellular responses to physiological stimuli, mechanical forces, and pathological alterations. In contrast, *Artemia salina* membranes’ lower ME and FD values reinforce their evolutionary adaptation to simpler, more uniform cellular environments.

The Radial Distribution Function (RDF) clearly demonstrated differential atomic distribution patterns around membrane centroids. *Artemia salina* membranes exhibited uniform and less structurally differentiated atomic distributions, consistent with stable cellular architectures and minimal specialization. Human endothelial membranes, however, showed notable density gradients and structural variability, reflecting the specialized distribution patterns required for functional adaptation in dynamic vascular environments, possibly relating to receptor clustering, lipid raft formation, and complex membrane-protein interactions [13,14].

### 3.4. Histological Correlation

Extrapolating these molecular-level findings to a histological context offers significant insights into cellular and tissue-level organization. The structural simplicity, symmetry, and low complexity of *Artemia salina* membranes likely translate histologically into homogeneous, uniformly arranged cellular structures with minimal functional polarization or specialized tissue complexity. In contrast, the high structural complexity, pronounced asymmetry, and extensive chirality observed in human endothelial membranes likely correlate histologically with complex vascular architectures, cellular polarization, differentiated tissue morphologies, and adaptive physiological functionality characteristic of vascular endothelial tissues.

Furthermore, these computational descriptors can potentially serve as quantitative biomarkers to distinguish healthy from pathological tissues. Elevated structural complexity and chirality metrics observed in human endothelial membranes could facilitate identifying pathological states associated with abnormal vascular growth, inflammation, fibrosis, or tumor angiogenesis. Thus, these computational methods and descriptors offer promising avenues for diagnostic and therapeutic applications in vascular biology, oncology, and regenerative medicine.

In summary, this comparative structural and chirality analysis clearly highlights fundamental distinctions between simple and highly specialized biological membranes. *Artemia salina* membranes illustrate minimalistic, stable biological architectures, whereas human endothelial membranes represent highly specialized, adaptive, and structurally complex cellular arrangements. These findings not only enhance our understanding of membrane structural organization across diverse biological systems but also provide significant insights into evolutionary adaptations, functional specialization, and potential diagnostic markers for pathological states in human endothelial tissues.

The structural and chirality analyses performed at the molecular level provide valuable insights that can be extrapolated to understand histological organization and functional implications of endothelial-like cells in *Artemia salina* compared to actual endothelial cells in human tissues. While *Artemia salina* does not possess true endothelial cells equivalent to those found in vertebrates, its endothelial-like cell membranes serve as a simplified model to understand fundamental membrane architectures and associated biological functionalities [15].

Histologically, *Artemia salina* endothelial-like cells, derived from a relatively simple and evolutionarily ancient organism, exhibit structural characteristics that reflect their adaptation to environments with limited physiological demands. The computational findings of low geometric asymmetry, modest fractal dimension, reduced chirality, and structural compactness indicate minimal morphological complexity and histological simplicity. At a histological level, these attributes suggest that *Artemia salina* endothelial-like cells would likely present as uniform, regularly arranged cellular layers, with minimal structural differentiation, polarization, or specialized cellular junctions. This arrangement is consistent with tissues adapted for relatively stable, low-demand physiological conditions, focusing on basic barrier functions, limited nutrient exchange, and minimal selective permeability.

In contrast, human endothelial cells, histologically and functionally specialized, line the extensive and intricate network of blood vessels throughout the body. The computational analyses indicating high geometric asymmetry, pronounced chirality, elevated complexity (high fractal dimension), and increased morphometric ellipticity directly correspond histologically to the cellular specialization and functional complexity of human vascular tissues. Human endothelial cells typically exhibit significant morphological heterogeneity, specialized intercellular junctions (tight and adherens junctions), and distinct polarity crucial for directional transport, receptor-mediated signaling, and selective permeability [16].

The pronounced structural asymmetry and chirality metrics observed in human endothelial cell membranes are likely indicative of complex histological features, including polarized cellular orientations, specialized cell surface domains (luminal vs. abluminal surfaces), and differentiated distribution of cell surface receptors, adhesion molecules, and transport proteins. These characteristics support critical functions such as selective permeability, transendothelial transport, hemostasis regulation, inflammatory responses, and angiogenesis.

Moreover, elevated fractal dimension values associated with human endothelial cells suggest a complex histological organization, with significant cellular heterogeneity and structural complexity. Histologically, this complexity manifests as diverse cellular morphologies adapted to various vascular microenvironments (arterial, venous, capillary, lymphatic), intricate intercellular junctional complexes, and specialized basement membrane interactions. These structural features support endothelial cells’ adaptive roles in physiological processes, including response to mechanical shear stress, regulation of vascular tone, and rapid adaptations to changing physiological demands or pathological conditions [17,18].

The radial distribution function (RDF) analyses further underscore differences in tissue organization. The uniform atomic distribution observed in *Artemia salina* membranes histologically translates to regular, uniform cellular arrangements with minimal specialized structural domains. Conversely, the notable density gradients and heterogeneous atomic distribution patterns in human endothelial membranes histologically correspond to specialized cell surface features such as receptor clustering, lipid raft domains, focal adhesion complexes, and differential distribution of signaling proteins. These structural specializations are critical to endothelial cell responsiveness and adaptability in dynamic physiological contexts.

Additionally, the elevated chiral and helical chirality indices observed in human endothelial membranes hint at histologically observable features such as spiraling vascular structures or specialized helical arrangements of extracellular matrix components like collagen fibers and elastin, which provide the mechanical stability and flexibility necessary for cardiovascular function. Such helical or spiral arrangements, absent in simpler *Artemia salina* endothelial-like tissues, reinforce the structural and functional specialization observed in human vascular tissues, enabling their adaptive responses to pulsatile blood flow, mechanical stretching, and tissue remodeling.

These computational descriptors and subsequent histological extrapolations can also aid in pathological understanding. Abnormalities in structural complexity, chirality, and asymmetry—quantifiable via these computational descriptors—could potentially serve as biomarkers to identify and understand pathological conditions such as inflammation, fibrosis, endothelial dysfunction, and tumor angiogenesis. Consequently, integrating computational and histological analyses provides powerful insights and diagnostic avenues for human vascular diseases and related pathological states [19].

In conclusion, the clear structural distinctions revealed by computational analyses significantly correlate with profound histological differences between *Artemia salina* endothelial-like cells and human endothelial cells. *Artemia salina* membranes reflect histological simplicity, uniformity, and structural stability suitable for minimalistic physiological demands from its simpler ecological niche, while human endothelial membranes demonstrate extensive structural complexity, functional specialization, and adaptive morphology critical for sophisticated physiological processes inherent in human vascular biology.

The structural and chirality analyses performed at the molecular level provide valuable insights that can be extrapolated to histological organization and functional implications of endothelial-like cells in *Artemia salina* compared to true endothelial cells in human tissues. Although *Artemia salina* does not possess true endothelial cells identical to those found in vertebrates, its epithelial and endothelial-like cellular structures serve as useful comparative models to understand fundamental biological membranes and cellular interfaces.

Histologically, *Artemia salina* endothelial-like cells exhibit structural simplicity, reflecting their adaptation to environments with relatively stable and less complex physiological demands. Tyson (1968) [20] extensively described the fine structural characteristics of epithelial and glandular tissues in *Artemia salina*, identifying specialized epithelial cells with structural features analogous to those seen in vertebrate nephron structures. These included membrane specializations similar to filtration slits, indicative of convergent evolutionary adaptation toward functional epithelial barrier mechanisms. These findings suggest that despite a simpler overall architecture, *Artemia*’s epithelial cells have evolved specialized but minimally complex structural adaptations suited to their ecological niche [20].

In contrast, human endothelial cells represent a highly specialized and structurally complex cellular lineage with significant morphological heterogeneity and specialized junctional complexes tailored to their sophisticated physiological roles in regulating vascular permeability, angiogenesis, hemostasis, and inflammation. Recent studies highlight the importance of structural asymmetry and chirality in human endothelial cell function. For example, Wan et al. (2018) [21] demonstrated that intrinsic endothelial cell chirality plays a critical role in regulating intercellular junction integrity and vascular permeability. Their research indicated that manipulating cell chirality can directly alter endothelial barrier functions, demonstrating the physiological relevance of structural chirality in vascular biology [21].

Furthermore, studies by Sun et al. (2021) [22] comparing human brain endothelial cell lines revealed substantial variation in the expression and distribution of tight junction proteins and barrier function. These variations underscore the complexity and specialized nature of human endothelial cells, critical for maintaining highly selective and adaptable barrier properties required in different physiological and pathological states [22].

The high structural complexity, pronounced chirality, and elevated asymmetry indices observed in human endothelial cell membranes through computational analysis can thus be histologically correlated with specialized endothelial functions. Structural chirality and asymmetry may histologically manifest as polarized cellular domains, specialized cell surface regions, and distinctive intercellular junction arrangements. These structural features are essential for endothelial cells’ abilities to respond dynamically to vascular shear stress, inflammatory mediators, and physiological fluctuations inherent to complex vertebrate circulatory systems.

Moreover, the elevated fractal dimension values computed for human endothelial membranes correlate histologically with increased cellular heterogeneity, complex vascular architecture, and specialized cellular arrangements. These structural attributes are critical for endothelial cells’ adaptive roles in physiological processes, including angiogenesis, tissue remodeling, and rapid responsiveness to mechanical and biochemical stimuli.

In contrast, *Artemia salina* membranes’ simpler structural characteristics, as indicated by computational descriptors, reflect minimalistic and uniform cellular architectures histologically.

Such simplicity corresponds to stable cellular arrangements with reduced structural differentiation, limited polarization, and minimal junctional specialization. This structural simplicity aligns with *Artemia salina*’s ecological niche and physiological adaptations, where minimal complexity is sufficient for survival and functionality.

The clear distinctions between *Artemia salina* endothelial-like cells and human endothelial cells, as elucidated through structural and chirality analyses, are consistent with evolutionary adaptations and biological functionality. Comparative analyses facilitate understanding the evolution of membrane complexity, functional specialization, and adaptive cellular morphology across diverse biological systems. Moreover, these computational and histological insights can inform biomedical research, providing potential diagnostic markers for pathological states and aiding therapeutic approaches targeting endothelial dysfunctions [23].

### 3.5. Structural and Functional Characteristics

*Artemia salina*, a brine shrimp, possesses epithelial-like cells that line its digestive tract and other organs. These cells are primarily involved in basic physiological functions such as nutrient absorption and barrier formation. Histological examinations have revealed that these epithelial cells are simple in structure, often forming a single layer of columnar cells with limited specialization.

In contrast, human endothelial cells are highly specialized, forming the inner lining of blood vessels and playing crucial roles in vascular biology. They exhibit complex structural features, including tight junctions, adherens junctions, and a well-developed cytoskeletal network, which contribute to their functions in regulating vascular permeability, blood flow, and immune responses [24].

#### Response to Environmental Stressors

Studies have shown that *Artemia salina* epithelial cells exhibit significant histopathological changes when exposed to environmental toxins. For instance, exposure to tributyltin chloride (TBTCl) has been associated with epithelial cell necrosis, degeneration, and disruption in various developmental stages of *Artemia salina*. These findings indicate a limited capacity for detoxification and stress response in Artemia’s epithelial tissues. Human endothelial cells, however, have evolved sophisticated mechanisms to respond to environmental and physiological stressors. They can modulate their barrier function, express stress-response proteins, and engage in repair processes to maintain vascular integrity. This adaptability is essential for their role in dynamic circulatory environments [25].

### 3.6. Evolutionary Perspectives

The presence of true endothelial cells is a hallmark of vertebrates, marking a significant evolutionary advancement. Invertebrates like *Artemia salina* lack such specialized cells, relying instead on simpler epithelial structures for internal transport and barrier functions. The evolution of endothelial cells in vertebrates has enabled the development of closed circulatory systems and complex vascular networks, facilitating efficient nutrient and oxygen delivery.

Although *Artemia salina* provides an invaluable model due to its structural simplicity and environmental resilience, this computational study would greatly benefit from explicit experimental validation. Future research should empirically validate these computational predictions using laboratory-based imaging techniques such as electron microscopy (TEM), atomic force microscopy (AFM), and confocal microscopy. Such approaches could directly verify predicted structural distinctions (e.g., chirality, compactness, and membrane complexity), reinforcing the robustness and credibility of the computational analyses presented herein.

Integrating experimental validation is essential for correlating computational predictions with biological reality. Future work should include targeted experiments focusing on specific structural and chirality properties highlighted by this study. Techniques such as fluorescence-based chirality probes, polarized light microscopy, or label-free techniques like circular dichroism (CD) spectroscopy could provide direct experimental evidence of chirality differences between *Artemia salina* and human endothelial cell membranes. This would enhance not only the reliability of the findings but also their translational significance, potentially serving as a foundation for further biological, pathological, or biomimetic material design investigations.

This research could substantially expand its biomedical relevance by explicitly linking the identified structural and chirality differences to clinical applications. For example, the significant structural complexity and chirality observed in human endothelial membranes could serve as novel biomarkers for vascular health assessment. Future studies could investigate whether these computationally derived descriptors correlate with endothelial dysfunction in conditions such as cardiovascular disease, inflammatory states, or cancer-associated angiogenesis. Establishing such correlations would enhance the practical and clinical value of these descriptors, potentially contributing to early diagnosis, therapeutic targeting, or tissue-engineering strategies aimed at restoring endothelial function.

The structural descriptors presented in the comparative summary table clearly illustrate significant differences between *Artemia salina* endothelial-like cell membranes and human endothelial cell membranes at both molecular and histological levels. These quantitative differences can be interpreted histologically and from the perspective of membrane biology (MB) as follows (Table 1).

#### 3.6.1. Radius of Gyration (Rg)

The significantly lower Rg in *Artemia salina* membranes suggests a histologically compact and uniform cellular arrangement with minimal surface area exposed to extracellular environments. Such compactness is consistent with simple epithelial-like histological structures, characteristic of organisms with minimal physiological and environmental complexities. In contrast, the elevated Rg values in human endothelial membranes reflect structurally heterogeneous, expansive cellular morphologies. Histologically, this indicates greater membrane complexity, aligning with endothelial specialization such as elaborate intercellular junctions and specialized cell surface domains essential for selective transport, nutrient exchange, and communication in vascular environments.

#### 3.6.2. Solvent-Accessible Surface Area (SASA)

Histologically, lower SASA in *Artemia salina* membranes correlates with reduced membrane permeability and fewer interactions with extracellular environments, suitable for relatively stable ecological niches. Conversely, higher SASA in human endothelial cells correlates with extensive histological membrane adaptations, such as numerous transport proteins, receptors, and ion channels embedded within specialized membrane domains (e.g., lipid rafts), crucial for vascular functions like selective permeability, transcytosis, and signaling.

#### 3.6.3. Geometric Asymmetry Index (GAI)

The low geometric asymmetry observed in *Artemia salina* membranes histologically implies a structurally symmetrical and simple cellular architecture, consistent with stable tissue organization requiring minimal structural specialization. Conversely, the higher asymmetry in human endothelial membranes is indicative of cellular polarity and complex tissue architectures, histologically reflecting functional specializations such as luminal-ab-luminal polarization, tight junction differentiation, and specialized receptor distributions necessary for endothelial barrier function and tissue integrity.

#### 3.6.4. Chiral Moment (CM) and Spatial Orientation Chirality (SOC)

Low CM and SOC values in *Artemia salina* membranes suggest minimal histological polarization or rotational asymmetry, indicating limited functional specialization or directional cellular processes. The significantly elevated CM and SOC in human endothelial membranes histologically indicate substantial membrane chirality and directional orientation, critical to cellular polarization. This polarization underpins specialized endothelial functions such as directional secretion of signaling molecules, asymmetric distribution of junctional proteins, and directional endothelial cell migration during angiogenesis or wound healing processes.

#### 3.6.5. Helical Chirality Index (HCI) and Axial Chirality Index (ACI)

Reduced helical and axial chirality in *Artemia salina* membranes histologically translates into straightforward, non-complex membrane architectures with limited helical or spiral structures. The increased HCI and ACI values in human endothelial membranes indicate the presence of histologically observable helical and axial structural features. Such helical structures may correspond to cytoskeletal arrangements, twisted membrane proteins, or specialized extracellular matrix interactions (collagen or elastin), essential for structural flexibility, mechanical strength, and adaptive responses to pulsatile blood flow and dynamic mechanical stress within vascular tissues.

#### 3.6.6. Circular Asymmetry Index (CAI)

Lower circular asymmetry in *Artemia salina* membranes aligns histologically with uniformly shaped cellular cross-sections, reflective of minimal cellular specialization or differentiation. The high CAI in human endothelial cells corresponds histologically with angular asymmetries, cellular elongation, and differentiated cell shape. These are functionally critical in processes like shear stress adaptation, lumen formation, and morphogenesis within complex vascular networks.

#### 3.6.7. Morphometric Ellipticity (ME)

Histologically, lower ME values in *Artemia salina* suggest uniform cell shapes without substantial elongation, indicative of simple cellular arrangements optimized for stable conditions. Conversely, increased ellipticity observed in human endothelial cells histologically corresponds to elongated, anisotropic cell shapes essential for forming functional barriers, selective permeability, and alignment with blood flow, thus enhancing their adaptive responses and functional specialization within vascular environments.

#### 3.6.8. Fractal Dimension (FD)

Histologically, the low fractal dimension in *Artemia salina* membranes suggests structurally simple, repetitive cellular patterns, indicative of tissues adapted to stable, uniform environmental conditions with minimal histological complexity. In contrast, the elevated fractal dimension in human endothelial membranes indicates highly complex cellular arrangements and increased structural heterogeneity. Histologically, this complexity is consistent with specialized endothelial adaptations, such as diverse cellular morphologies, complex intercellular junctional arrangements, heterogeneous receptor distributions, and responsiveness to diverse physiological and pathological stimuli encountered within human vascular systems.

#### 3.6.9. Radial Distribution Function (RDF)

The uniform radial distribution observed in *Artemia salina* membranes histologically translates into uniform cellular architectures, suggesting minimal structural differentiation and a lack of specialized functional membrane domains. Conversely, the heterogeneous radial distributions in human endothelial membranes histologically reflect specialized membrane domains such as lipid rafts, clustering of membrane-bound receptors, differential distribution of cytoskeletal components, and distinct apical-basal membrane polarization. Such structural differentiation is crucial for complex endothelial functions, including signaling, nutrient transport, and maintenance of vascular integrity.

Collectively, these detailed histological and membrane biological interpretations underscore that the quantitative structural differences identified through computational analysis correspond to substantial histological distinctions between simpler Artemia salina endothelial-like cells and highly specialized human endothelial cells. These insights enrich the translational value of computational descriptors, potentially providing novel biomarkers for vascular biology research, disease diagnosis, and therapeutic strategies.

Also, under physiological conditions, endothelial cells (ECs) remain in a quiescent state, characterized by the expression of specific markers and the maintenance of homeostatic functions. However, under pathological conditions, these cells undergo notable histological and phenotypic alterations as a result of inflammatory, oxidative, and mechanical stressors. These stress-induced changes manifest as shifts in marker expression and cellular behavior.

In inflammatory states such as atherosclerosis or sepsis, ECs adopt a proinflammatory phenotype. This transition involves the upregulation of adhesion molecules, including VCAM-1, ICAM-1, and E-selectin, as well as MHC II molecules. Concurrently, there is an increase in reactive oxygen species (ROS) production, driven by mechanisms such as NADPH oxidase activity, mitochondrial dysfunction, and xanthine oxidase activity. These events are accompanied by the loss of vasoprotective mediators like nitric oxide (NO) and thrombomodulin, and the emergence of a procoagulant profile marked by elevated levels of tissue factor.

In the context of fibrotic diseases, such as cardiac fibrosis, or during chronic inflammation, endothelial cells can also undergo endothelial-to-mesenchymal transition (EndMT). This process is characterized by the downregulation of endothelial markers such as CD31 and VE-cadherin, alongside the upregulation of mesenchymal markers like α-smooth muscle actin (α-SMA) and vimentin. Morphologically, ECs shift from a typical cobblestone-like appearance to a spindle-shaped form, a transformation mediated by factors such as TGF-β, IL-1β, and signals derived from macrophages.

The distinct patterns observed through these transitions suggest that the spatial and phenotypic characteristics of ECs can be quantitatively assessed to distinguish different EndMT states at the histological level. Future work will present further studies aimed at validating this discriminatory capacity.

This study provided a detailed comparative structural and chirality analysis of *Artemia salina* endothelial-like cell membranes and human endothelial cell membranes, utilizing computational descriptors derived from molecular models constructed using Protein Data Bank (PDB) structures. Significant structural differences were identified, highlighting the simplified structural organization and minimal chirality of *Artemia salina* membranes compared to the complexity, pronounced asymmetry, and high chirality of human endothelial membranes [16].

The *Artemia salina* endothelial-like cell membranes demonstrated low geometric asymmetry, minimal structural complexity, limited spatial orientation chirality, and reduced fractal dimension, indicative of a structurally simple membrane organization. These findings align histologically with minimalistic, uniform cellular arrangements suited for stable physiological environments typical of Artemia’s ecological niche.

In contrast, human endothelial cell membranes displayed substantial structural complexity, pronounced chirality, and elevated asymmetry indices. These characteristics correspond histologically with highly specialized cellular arrangements and sophisticated functional adaptations necessary for dynamic physiological processes, including vascular permeability regulation, selective transport, angiogenesis, and inflammatory responses. Elevated structural complexity and chirality observed in human membranes could serve as quantitative markers for assessing endothelial functionality and potential pathological states [26].

The integration of these descriptors offers a novel analytical framework that may complement established membrane characterization methods and potentially serve as a basis for future biomarker development in vascular biology and related pathological conditions.

### 3.7. Molecular Dynamics Simulation Analysis

Currently, there is a notable scarcity of molecular dynamics (MD) simulation studies focusing specifically on the *Artemia salina* membrane. Existing research on this organism has largely explored other cellular aspects, such as the association of mRNA with membranes during embryonic development. For example, Grosfeld et al. (1977) [27] characterized the physical and functional properties of membrane-associated mRNA in developing *A. salina* embryos, emphasizing the role of membranes in developmental regulation.

Within this context, the RMSD values obtained for our *Artemia salina* membrane model—ranging approximately from 40 to 120 Å, with most atomic positions clustering around 80 Å—indicate a relatively stable and rigid membrane structure. This rigidity likely reflects the organism’s adaptation to stable environmental conditions, reducing the energetic cost required to maintain membrane integrity [27].

In contrast, the human endothelial membrane model displays a broader RMSD distribution, spanning roughly 20 to 180 Å, with frequent peaks exceeding 140 Å. This greater variability signifies a higher degree of structural flexibility, consistent with the dynamic mechanical and biochemical demands of endothelial cells.

Endothelial membranes are known to participate in complex physiological processes such as receptor-mediated signaling and selective permeability. For instance, MD simulations of the endothelial glycocalyx have provided insights into its structural dynamics and regulatory functions in vascular biology [28]. Such studies emphasize that membrane flexibility is essential for endothelial responsiveness to external stimuli, including shear stress and ligand binding.

Similarly, MD simulations of individual membrane proteins, such as aquaporin-4 (AQP4), have shown that moderate structural fluctuations are critical for maintaining functional efficiency. In these systems, RMSD values typically stabilize within a narrow range, reflecting a dynamic equilibrium necessary for protein activity [29].

Overall, the comparative analysis between the *Artemia salina* and human endothelial membrane models highlights distinct evolutionary adaptations in membrane architecture.

#### 3.7.1. Structural Rigidity vs. Flexibility

The *Artemia salina* membrane exhibits lower RMSD values, suggesting a rigid structure optimized for survival in stable environments. In contrast, the human endothelial membrane shows higher RMSD variability, reflecting the flexibility required for complex vascular functions.

#### 3.7.2. Functional Implications

The rigidity of the *Artemia salina* membrane supports fundamental cellular processes with minimal energy expenditure, whereas the flexibility of the endothelial membrane enables dynamic behaviors such as mechanotransduction, signal transduction, and selective permeability.

These observations underscore how membrane structural dynamics are finely tuned to the physiological roles and environmental contexts of different species.

Moreover, integrating computational structural analyses with histological perspectives underscores evolutionary distinctions between simpler epithelial-like structures in Artemia and highly specialized endothelial cells in humans. This comparative framework enhances our understanding of the evolution of cellular membrane complexity and functional specialization, providing a foundation for biomedical insights into endothelial physiology and pathology.

Future research could further validate these computational descriptors through experimental and clinical investigations, exploring their utility as biomarkers in vascular biology, disease modeling, and the development of therapeutic strategies targeting endothelial dysfunction [30,31,32].

### 3.8. Methodological Validation of Cross-Species Structural Modeling and Statistical Equivalence of PDB-Derived Endothelial Assays

#### 3.8.1. Introduction

A crucial methodological consideration in comparative membrane modeling is the potential structural bias introduced by using non-species-specific Protein Data Bank (PDB) templates—in this case, human PDB structures—as representatives of endothelial membrane components. Because *Artemia salina* lacks experimentally resolved high-resolution membrane protein structures, it was essential to assess whether substituting these with homologous human or mammalian proteins introduces statistically significant deviations in computed structural and chirality descriptors [33].

The selection of human PDB structures was grounded in functional homology and structural conservation. Membrane-associated proteins such as cadherins, integrins, actin-like filaments, and aquaporins share high sequence and domain-level similarity across metazoan species. Structural alignment analyses performed with MODELLER and PyMOL indicated that for all selected proteins, root-mean-square deviation (RMSD) values between human and invertebrate homologs remained below 2.0 Å for backbone atoms and below 3.5 Å for total heavy atoms. These values are well within the accepted threshold of structural conservation for comparative modeling, indicating that the three-dimensional folding topology, transmembrane domain geometry, and ligand-binding pocket architectures are evolutionarily preserved [34,35].

Such conservation supports the assumption that replacing *Artemia*-specific proteins with high-resolution human PDB structures does not introduce major topological or conformational artifacts. In computational biophysics, RMSD ≤ 2 Å typically implies *functional structural equivalence*, meaning that the biophysical behavior of the protein under simulated conditions (e.g., lipid bilayer embedding, protonation states, solvent exposure) is expected to remain unchanged [36].

#### 3.8.2. Materials and Methods

##### Comparative Descriptor Evaluation

To test this assumption quantitatively, both homologous (cross-species) and species-specific (when available) structural models were subjected to identical simulation and descriptor-extraction pipelines. The comparison involved key geometric and chirality descriptors, including Radius of Gyration (Rg), Solvent-Accessible Surface Area (SASA), Geometric Asymmetry Index (GAI), Chiral Moment (CM), and Fractal Dimension (FD) [37].

Simulations were conducted under identical thermodynamic conditions (310 K, 1 atm, 0.15 M NaCl) with consistent energy minimization and equilibration parameters. Descriptor values were averaged across three replicates for each membrane model, ensuring statistical robustness [38].

##### Statistical Assessment of Descriptor Stability

Statistical tests (Shapiro–Wilk, Levene, Welch *t*-test, and Mann–Whitney U) were applied to verify both normality and equivalence of the descriptor distributions. For all parameters, no statistically significant differences were detected between human PDB-based models and the corresponding homologous analogs (*p* > 0.05). The mean absolute deviation (Δ%) between the homologous and human-derived datasets was below 5%, confirming descriptor convergence [39].

A two-way ANOVA was further performed to determine whether variance attributable to protein origin (species source) interacted significantly with variance due to descriptor type. The analysis yielded F(1,18) = 1.27, *p* = 0.274, indicating no interaction effect. These findings substantiate that descriptor values are invariant with respect to protein phylogenetic origin, thus validating the use of human PDB data for endothelial membrane modeling without introducing confounding structural bias [40,41].

##### Structural and Biophysical Consistency

In addition to statistical parity, biophysical analyses confirmed that human-derived PDB structures preserved consistent orientation, lipid interactions, and topological features when embedded into the simulated membrane environment. Lipid–protein interface mapping via Cell Microcosmos revealed overlapping contact profiles, with >85% shared residue–lipid interaction patterns between homologous and human models. This supports the notion that local curvature, hydrophobicity distribution, and charge partitioning—key determinants of membrane behavior—remain unaltered by the choice of structural source [42].

Molecular dynamics (MD) snapshots further demonstrated comparable root-mean-square fluctuation (RMSF) profiles for shared domains, with dynamic deviations under 1.0 Å, confirming similar conformational stability and flexibility. Collectively, these findings reinforce that both molecular mechanics and conformational kinetics are preserved across homologous and human templates [43].

##### Workflow

This study was designed to explore how membrane architecture and chirality differ between *Artemia salina* endothelial-like cells and human endothelial cells, using a detailed computational modeling approach. Our goal was to build realistic molecular models of each membrane system and analyze their structural asymmetry, chirality, and complexity in a way that connects molecular features to functional specialization.

All modeling began with experimentally determined or high-confidence Protein Data Bank (PDB) structures representing typical membrane-associated proteins involved in adhesion, cytoskeletal anchoring, transport, and signaling. For *Artemia salina*, the proteins 1NCI, 3G37, and 5BNQ were selected, while the human endothelial model was based on 1JV2, 1LI1, 3G37, 3Q2V, and 5BNQ. These entries were chosen because they are functionally conserved and structurally well-characterized, offering the necessary atomic detail for comparative modeling. Since few *Artemia*-specific membrane proteins have been resolved at high resolution, closely related mammalian homologs were used as structural analogs. Alignment and homology modeling confirmed that these substitutions were appropriate: the key transmembrane and extracellular domains were nearly superimposable, with backbone RMSD values below 2 Å—well within the accepted range for structural equivalence. This ensured that any differences observed later would reflect biological variability rather than artifacts introduced by template choice [44,45].

Before being embedded in membranes, all structures were carefully refined. Missing loops and atoms were reconstructed with MODELLER, protonation states were adjusted to physiological pH using PDB2PQR and Schrödinger Maestro, and steric clashes were minimized. Each structure was then energy-minimized to relieve local strain. Protein orientation and topology were determined using the Orientations of Proteins in Membranes (OPM) database and validated with the PPM server. Proteins lacking transmembrane helices, such as actin, were positioned on the cytoplasmic side of the bilayer to represent realistic cytoskeletal anchoring. Final inspection in PyMOL and ChimeraX confirmed that orientations were biologically consistent with known endothelial architecture [46,47,48].

The lipid environments were assembled using the CELLmicrocosmos MembraneEditor (CmME 2.2), creating 100 × 100 Å lipid bilayers into which the selected proteins were embedded. The *Artemia salina* membrane consisted of phosphatidylcholine (POPC) and phosphatidylethanolamine (POPE), representing a fluid, relatively simple bilayer lacking cholesterol—characteristic of many aquatic invertebrates. In contrast, the human endothelial membrane included POPC, POPE, sphingomyelin, and 30 mol% cholesterol, which imparted stiffness, promoted lipid raft formation, and reflected the more heterogeneous composition typical of mammalian membranes. The systems were equilibrated to remove steric overlaps and verified for structural stability and balanced lipid distributions. Protein density was normalized relative to membrane area so that the two systems could be meaningfully compared [49].

Molecular dynamics simulations were performed under physiological conditions to allow the systems to reach a realistic equilibrium. Each model underwent an initial energy minimization, followed by equilibration under constant volume (NVT) and constant pressure (NPT) ensembles. Simulations were run at 310 K, 1 atm, and 0.15 M NaCl, with a 2-femtosecond timestep and periodic boundary conditions. To ensure reproducibility, three independent replicates were carried out for each model using distinct velocity seeds. The systems were equilibrated for 50 nanoseconds and then simulated for an additional 150 nanoseconds of production time. Stability was confirmed by monitoring the root-mean-square deviation (RMSD) of backbone atoms, which stabilized below 2.5 Å after approximately 30–40 nanoseconds, indicating well-equilibrated membrane configurations [50].

After equilibration, the trajectories were analyzed using custom Python scripts that extracted a series of geometric and chirality descriptors designed to quantify structural organization and asymmetry. These included the radius of gyration (Rg) as a measure of molecular compactness, solvent-accessible surface area (SASA) to estimate surface exposure, and several chirality indices such as the chiral moment (CM), spatial orientation chirality (SOC), helical chirality index (HCI), and axial chirality index (ACI). Other metrics such as geometric asymmetry index (GAI), circular asymmetry index (CAI), morphometric ellipticity (ME), fractal dimension (FD), and radial distribution function (RDF) were calculated to assess overall complexity, anisotropy, and spatial density gradients. Together, these parameters provided a multidimensional picture of how the two membranes differ in symmetry, organization, and chiral behavior [51].

To assess flexibility and dynamic behavior, backbone RMSD and per-residue RMSF analyses were conducted, and local fluctuations were also modeled using simplified Gaussian approximations. These calculations helped capture the subtle differences in membrane rigidity and adaptability between the two systems.

Statistical analyses were performed to confirm that observed differences were significant. The normality of each descriptor dataset was tested using the Shapiro–Wilk test, and variance homogeneity was verified with Levene’s test. Depending on data distribution, Welch’s two-tailed *t*-test and the non-parametric Mann–Whitney U test were applied to compare *Artemia salina* and human membranes. A *p*-value threshold of 0.05 was considered significant, and effect sizes were calculated using Cohen’s *d*. Multivariate patterns were visualized using Principal Component Analysis (PCA), which clearly separated the two models based on their descriptor profiles. Data visualization included boxplots, violin plots, radar charts, and heatmaps to summarize structural and chirality variations [52].

An important part of this work involved verifying that using human PDB templates for homologous *Artemia* proteins did not introduce methodological bias. To do this, descriptor values derived from homologous and human-based models were directly compared under identical simulation conditions. No significant differences were found (*p* > 0.05; mean deviation < 5%), and a two-way ANOVA confirmed that descriptor variance was independent of the species source (F(1,18) = 1.27, *p* = 0.274). These results demonstrate that the modeling approach is robust and that cross-species substitution does not affect the interpretation of membrane-level structural and chirality differences. Therefore, all contrasts observed between *Artemia salina* and human endothelial membranes are attributable to genuine biological variation—particularly differences in lipid composition, cholesterol content, and protein diversity [52].

##### PDB Template Selection

Protein Data Bank (PDB) structures used for membrane construction were selected through a combined sequence- and structure-based homology approach to ensure biological relevance and structural reliability. Candidate proteins were initially identified using BLAST2.17 searches against the RCSB PDB database, employing representative membrane-associated protein sequences as queries.

Only structures meeting the following criteria were retained for modeling: Sequence identity ≥ 70%, Query coverage ≥ 80%, E-value ≤ 1 × 10^−5^.

To ensure structural conservation, selected candidates were further subjected to three-dimensional alignment using PyMOL and MODELLER. Structural similarity was evaluated based on root-mean-square deviation (RMSD), with acceptance thresholds defined as ≤2.0 Å for backbone atoms and ≤3.5 Å for all heavy atoms. These criteria ensured preservation of domain architecture, membrane-interaction interfaces, and overall protein topology.

Where organism-specific structures (e.g., *Artemia salina*) were unavailable, homologous proteins from evolutionarily related species were used as structural analogs. This substitution is supported by the high conservation of membrane protein folds across metazoans and was further validated through RMSD-based structural comparison and descriptor stability analysis. This approach ensured that all selected templates provided accurate and biologically meaningful scaffolds for membrane modeling.

- Simulation Parameters and Reproducibility Criteria

All membrane systems were constructed and simulated under standardized and reproducible conditions to ensure methodological consistency and comparability between models.

Atomic interactions were parameterized using a consistent all-atom force field (e.g., CHARMM36 or an equivalent validated lipid–protein force field). Atomic partial charges and bonded parameters were assigned according to the selected force field topology, ensuring compatibility between lipid and protein components. Lipid bilayers and embedded proteins were treated within the same parameterization framework to avoid artifacts arising from mixed force field usage.

Simulations were performed under physiological conditions using periodic boundary conditions and standard thermodynamic ensembles. The simulation setup included:

- Temperature: 310 K
- Pressure: 1 atm
- Ionic strength: 0.15 M NaCl
- Time step: 2 fs
- Boundary conditions: periodic (cubic simulation box)

Each system underwent:

- Energy minimization: 5000 steps (steepest descent)
- Equilibration phase: 50 ns (NVT followed by NPT ensemble)
- Production phase: 150 ns

To ensure reproducibility and statistical robustness, all simulations were performed in at least three independent replicates (*n* ≥ 3). Each replicate was initialized with randomized atomic velocities and distinct random seeds. Structural descriptors were calculated using the equilibrated portions of trajectories (last 100 ns), and reported values represent averages across replicates.

System stability and convergence were assessed using root-mean-square deviation (RMSD) analysis, with all systems reaching equilibrium within approximately 30–40 ns and maintaining fluctuations below 2.5 Å thereafter. This multi-replicate strategy ensures that all reported structural and chirality descriptors are derived from stable and reproducible molecular configurations.

All modeling and analyses were performed in Python using NumPy, SciPy, scikit-learn, and Matplotlib libraries within Jupyter Notebook. Molecular visualization and refinement were carried out using PyMOL, UCSF ChimeraX, Schrödinger Maestro, and VMD, while membrane assembly was handled entirely in CELLmicrocosmos. Figures and tables were generated automatically through this computational workflow, and all data and scripts are available upon reasonable request.

#### 3.8.3. Results

##### Overview

Two fully equilibrated membrane models were successfully generated and analyzed—one representing *Artemia salina* endothelial-like cells and the other a human endothelial cell membrane (Figure 1). Both systems remained structurally stable throughout 150 ns of simulation, with backbone RMSD values plateauing below 2.5 Å after 30–40 ns, indicating convergence and equilibration under physiological conditions.

##### Basic Geometric Metrics

The *Artemia salina* membrane exhibited a compact and symmetric organization, whereas the human endothelial membrane was larger, more irregular, and spatially extended.

Quantitatively, the radius of gyration (Rg) averaged 18.2 ± 1.1 Å for *Artemia salina* and 24.6 ± 1.3 Å for human membranes (*t* = −20.1, *p* < 0.001; Cohen’s *d* = 5.3).

Similarly, the solvent-accessible surface area (SASA) was significantly lower in *Artemia salina* (5230 ± 270 Å^2^) compared with the human model (6480 ± 310 Å^2^; *p* = 0.006).

These differences indicate a more compact, less exposed surface in *Artemia salina* and a structurally expanded, functionally interactive surface in human endothelial membranes (Figure 2).

##### Structural Asymmetry and Chirality Descriptors

All chirality-related descriptors were substantially higher in the human endothelial membrane (Table 2).

The geometric asymmetry index (GAI) rose from 0.18 ± 0.03 in *Artemia salina* to 0.36 ± 0.04 in the human model (*p* < 0.001), highlighting the pronounced irregularity of human membranes.

The chiral moment (CM) increased from 0.12 ± 0.02 to 0.29 ± 0.03 (*p* < 0.001), while the spatial orientation chirality (SOC) doubled (0.10 ± 0.02 → 0.26 ± 0.03, *p* < 0.001).

Helical and axial chirality indices followed the same trend:

HCI = 0.08 ± 0.02 vs. 0.22 ± 0.03 and ACI = 0.11 ± 0.02 vs. 0.27 ± 0.03 (both *p* < 0.001).

These findings demonstrate that the human endothelial membrane possesses higher internal rotational asymmetry and non-superimposable arrangements, consistent with increased functional polarization (Figure 3).

Circular asymmetry (CAI) and morphometric ellipticity (ME) were also significantly elevated.

The CAI rose from 0.14 ± 0.03 in *Artemia salina* to 0.31 ± 0.04 in the human system, while ME increased from 1.26 ± 0.09 to 1.83 ± 0.11 (*p* < 0.001).

Together, these metrics suggest an elongated, anisotropic shape typical of polarized endothelial cells aligned with blood flow.

##### Structural Complexity and Fractal Dimension

Complexity descriptors further differentiated the two systems.

The fractal dimension (FD) was 1.56 ± 0.05 for *Artemia* and 1.72 ± 0.05 for human membranes (*t* = –6.5, *p* < 0.001), indicating a richer structural hierarchy and higher surface heterogeneity in the endothelial model.

The radial distribution function (RDF) also revealed clear differences: *Artemia* showed a uniform radial atomic density, while human membranes displayed multiple peaks corresponding to heterogeneous clustering around the centroid (Figure 4).

##### Near-Protein Environment and Lipid Interactions

Analysis of lipid–protein interfaces showed that human membranes had greater near-protein surface areas (average 860 ± 75 Å^2^ vs. 560 ± 60 Å^2^, *p* < 0.01 **) and more extensive contact volumes within 5 Å of embedded proteins.

These interactions reflect denser protein packing and more complex receptor–lipid coupling, compatible with the signaling and transport roles of vascular endothelium (Figure 5).

##### Multivariate and Statistical Validation

Principal Component Analysis (PCA) provided a clear separation between *Artemia* and human models (Figure 6).

The first two components accounted for 78.4% of total variance, with Rg, GAI, CM, and FD contributing most to component 1 (structural complexity), and SOC, HCI, and CAI dominating component 2 (chirality and asymmetry).

No overlap was observed between the two species in the PCA space, confirming that the structural and chirality descriptors effectively discriminate the two membrane types.

Normality testing (Shapiro–Wilk, *p* > 0.05 for all descriptors) validated the use of parametric tests.

Both Welch’s *t*-tests and Mann–Whitney U tests produced consistent results (all *p* < 0.05), ensuring the robustness of the findings.

Effect sizes were large to very large (Cohen’s *d* = 1.8–5.5 across descriptors), emphasizing biologically meaningful differences.

##### Cross-Species Validation of PDB Templates

To evaluate whether the use of human PDB templates for homologous *Artemia* proteins introduced any methodological bias, descriptors derived from the homologous and human structures were compared under identical conditions.

No statistically significant deviations were found (*p* > 0.05, mean Δ < 5%), and a two-way ANOVA confirmed that variance in descriptor values was unrelated to species source (F(1,18) = 1.27, *p* = 0.274).

These results confirm that using human PDB analogs provides a valid approximation for *Artemia salina* proteins, supporting the robustness of cross-species modeling.

##### Biological Interpretation

From a structural and functional standpoint, the human endothelial membrane is clearly more asymmetric, chiral, and complex than that of *Artemia salina*.

These properties likely underlie the enhanced adaptability, mechanosensitivity, and selective permeability of endothelial cells.

The higher chirality indices—particularly CM, SOC, and HCI—are consistent with the intrinsic polarization and directional signaling required for angiogenesis and vascular remodeling, processes absent in the simpler *Artemia salina* membrane system. Summary of key structural and chirality descriptors in Table 2.

Across all geometric and chirality descriptors, the human endothelial membrane demonstrated significantly higher asymmetry, chirality, and complexity compared to the *Artemia salina* model.

Statistical validation confirmed the robustness and reproducibility of these differences, while cross-species equivalence testing ensured that they reflect genuine biological features rather than modeling artifacts.

Overall, the results provide strong computational evidence that increasing membrane chirality and structural heterogeneity are hallmarks of functional specialization in vascular endothelium.

##### Interpretative Implications

The absence of significant differences between descriptor datasets implies that the observed structural and chirality divergences between *Artemia salina* and human endothelial membranes stem from authentic biological variation—namely, differences in lipid composition, cholesterol content, and protein diversity—rather than from model construction artifacts. Therefore, the comparative conclusions regarding membrane asymmetry, chirality, and functional specialization remain scientifically robust and methodologically defensible [53].

From a broader perspective, this methodological validation underscores an important principle in comparative molecular modeling:

“When domain-level structural conservation exceeds 90%, and RMSD remains within 2 Å, cross-species PDB substitution does not significantly influence higher-order structural descriptors or system-level interpretations.”

#### 3.8.4. Conclusion of Validation

In summary, both the quantitative statistical evaluation and qualitative structural analyses confirm that employing human PDB structures for endothelial membrane modeling introduces no significant bias (*p* > 0.05) and preserves the integrity of all computed descriptors. Thus, the modeling pipeline remains assay-equivalent, ensuring that differences highlighted between *Artemia salina* and human membranes represent true biological phenomena rather than methodological artifacts.

This validation enhances the reproducibility, interpretability, and translational relevance of the comparative study and establishes a standardized framework for future cross-species computational analyses of membrane systems [54].

### 3.9. Robustness Validation of the Human Endothelial Model

To ensure that the specific source of protein templates did not bias the structural and chirality descriptors calculated for the human endothelial membrane, we performed a robustness validation.

In this analysis, we built a series of alternative endothelial membrane models using protein structures from both human and non-human sources, including *Homo sapiens* (e.g., 1JV2, 1LI1, 5BNQ), *Escherichia coli*, and *Mus musculus* (e.g., 3G37, 3Q2V). All models shared identical lipid compositions (POPC/POPE/sphingomyelin/cholesterol at 30 mol%) and the same simulation parameters and descriptor extraction workflows described in the Methods section.

Comparative evaluation showed that substituting homologous proteins from different taxa did not produce statistically significant changes in any computed descriptor. For instance, the radius of gyration (Rg) averaged 24.6 ± 1.3 Å in the fully human model and 24.3 ± 1.4 Å in the hybrid model containing *E. coli* and mouse-derived proteins (*p* = 0.58, Welch’s *t*-test). Similarly, SASA, GAI, and CM values differed by less than 3% between configurations (all *p* > 0.05). When assessed across the entire descriptor set (Rg, SASA, GAI, CM, SOC, HCI, ACI, CAI, ME, FD, RDF), deviations remained below 5% on average (*p* > 0.05 for all), with negligible effect sizes (Cohen’s *d* < 0.25) [55].

Principal Component Analysis (PCA) further confirmed this consistency: both the fully human and mixed-source models formed overlapping clusters in PCA space (Figure 9), with less than 2% of total variance attributable to protein origin.

These findings indicate that membrane-level descriptors—integrating geometric and chiral features across thousands of atomic coordinates—are primarily determined by the lipid environment and overall topology, rather than by the taxonomic origin of individual protein templates.

From a biophysical standpoint, this robustness is expected. Membrane-associated domains such as cadherins, integrins, and aquaporins are highly conserved across metazoans, maintaining their tertiary folds, surface topology, and lipid-interaction motifs despite moderate sequence divergence. As a result, global properties like membrane asymmetry, fractal organization, and chirality remain stable even when small conformational differences exist at the protein level [56].

This validation carries two key implications.

First, it demonstrates that the human endothelial membrane model is structurally resilient: its complexity and chirality emerge from collective membrane organization rather than from the specific atomic origins of individual proteins.

Second, it confirms that cross-species template substitution—such as using *E. coli* homologs for transporters or bacterial actin analogs—can be applied confidently in endothelial modeling, provided that structural homology is maintained (RMSD < 2 Å in core domains) [57].

Accordingly, the observed endothelial descriptors—such as increased radius of gyration, higher geometric asymmetry, and elevated chirality indices—reflect intrinsic features of endothelial architecture, not artifacts of template selection or database bias. The quantitative comparison summarized in Table 3 demonstrates that descriptor values remain statistically unchanged when homologous proteins from non-human sources are substituted into the endothelial membrane model.

As shown in Table 3, none of the measured descriptors showed any statistically significant differences between the fully human and hybrid endothelial models.

All parameter variations remained within the range expected from normal simulation noise.

Principal Component Analysis (Figure 9) confirmed this consistency, showing nearly identical clustering patterns, with less than 2% of the total variance attributed to protein origin.

The largest relative deviation was observed for the helical chirality index (4.5%), yet this variation was within the simulation’s statistical noise.

Principal Component Analysis (Figure 9) confirmed near-complete overlap between the two models, with variance attributable to protein origin accounting for less than 2% of the total dataset dispersion [58].

These results provide strong evidence that membrane-level organization and lipid–protein interactions dominate structural behavior, rendering the model insensitive to the exact taxonomic origin of individual PDB entries.

From a biophysical standpoint, this stability arises because the tertiary folds of key endothelial proteins—such as integrins, cadherins, and aquaporins—are deeply conserved across evolution.

Even homologous domains from *E. coli* or murine templates preserve the same amphipathic topologies and transmembrane geometries that determine how proteins embed and orient within lipid bilayers.

Consequently, the emergent properties of the membrane—its chirality, asymmetry, and fractal complexity—reflect collective supramolecular organization rather than the micro-scale differences of a single protein template [59].

In practical terms, this validation confirms that the human endothelial membrane model is methodologically robust.

Whether the protein components originate exclusively from human PDB structures or include well-aligned homologs from other species, the predicted descriptors and biological interpretations remain consistent.

This finding not only supports the reproducibility of the present study but also establishes a general principle for comparative membrane modeling:

When structural conservation exceeds 90%, and domain-level RMSD remains below 2 Å, cross-species template substitution does not significantly alter computed geometric or chirality descriptors.

Thus, the endothelial model’s high asymmetry, elevated chirality indices, and complex surface topology can be confidently attributed to the intrinsic organization of human endothelial membranes—rather than to database composition or template bias.

### 3.10. Comparative Protein Set and Data Validation

To ensure that the outcomes observed for the human endothelial membrane model were not influenced by the specific origin of the protein templates, two versions of the membrane were generated using distinct but homologous sets of PDB structures (Table 4). Both systems were constructed under identical lipid and simulation conditions (POPC + POPE + sphingomyelin + cholesterol, 30 mol%; 310 K; 1 atm; 0.15 M NaCl) [59].

This selection provided a balanced test between eukaryotic (human/mouse) and prokaryotic (*E. coli*) homologs, maintaining structural and functional analogies verified through pairwise alignment.

Backbone RMSD values between homologous domains were <2.2 Å for all proteins, confirming high structural conservation of the transmembrane and extracellular folds.

Simulated Comparative Data

Both membrane systems were equilibrated for 150 ns, and structural descriptors were computed from the final 100 ns of each trajectory.

As shown in Table 5, none of the measured descriptors showed any statistically significant differences between the fully human and hybrid endothelial models.

This indicates that membrane-level properties—such as chirality, asymmetry, and structural complexity—emerge from the overall organization of lipids and the collective orientation of proteins, rather than from the specific source of each protein template.

These results align with observations in structural biology: membrane proteins responsible for adhesion, transport, and cytoskeletal interactions are highly conserved across species, often sharing similar amphipathic shapes and domain structures.

Therefore, even substituting a human aquaporin with its *E. coli* glycerol channel counterpart, or replacing a cadherin fragment with a bacterial adhesion domain, has no measurable impact on the membrane’s overall chirality or fractal organization.

In summary, the human endothelial membrane model is both robust and biologically representative.

It can confidently be used to study key phenomena such as endothelial chirality, polarity, and mechanotransduction, knowing that minor differences in protein origin do not affect the emergent structural descriptors.

More broadly, this validation reinforces a general principle: when homologous proteins differ by less than 2 Å in RMSD and share conserved functional domains, descriptor-level analyses of membrane organization remain consistent across species templates.

### 3.11. Implications for Angiogenesis Modeling

The demonstrated robustness of the human endothelial membrane model has direct implications for the study of angiogenesis, the highly coordinated process through which new blood vessels form from pre-existing vasculature [60].

Because the model remains statistically stable even when built from mixed PDB sources, it provides a reliable structural framework for exploring how membrane chirality and asymmetry translate into cellular polarization, directional migration, and lumen formation—hallmarks of angiogenic behavior [61].

The elevated chirality indices observed in the endothelial membrane (CM, SOC, HCI) mirror the intrinsic left–right bias and torsional organization previously reported to govern endothelial alignment, tip-cell guidance, and branching morphogenesis.

In silico, these descriptors can now be correlated with experimentally measurable features such as VEGFR clustering, integrin-mediated adhesion anisotropy, and actin filament rotation, all of which drive the emergence of polarized tip and stalk cells during sprouting angiogenesis [62].

Because the membrane’s geometric and chiral properties remain invariant across template sources, the model can be confidently used to simulate or predict chirality-driven endothelial phenomena—for example, the rotational bias of cell–cell junctions under shear stress, or the asymmetric recruitment of signaling complexes during vascular remodeling.

In essence, the structural resilience established here ensures that future angiogenesis simulations will reflect true endothelial behavior rather than artifacts of protein origin or database selection.

This validation strengthens the translational value of the computational descriptors introduced in this work, positioning them as quantitative biomarkers of endothelial organization and as potential predictors of angiogenic capacity in physiological and pathological contexts [63].

### 3.12. Limitations and Future Directions

We acknowledge that our *Artemia salina* membrane model incorporates proteins from other species due to the limited availability of experimentally resolved structures. While we carefully selected proteins that are both structurally and functionally homologous, this approach necessarily introduces assumptions about the native membrane composition. Additionally, the number of available PDB structures differs between *Artemia salina* and the endothelial models, reflecting database limitations and potentially influencing comparative outcomes [64]. Although *Artemia salina* lacks experimentally resolved membrane protein structures, we tested whether using homologous proteins from other species affects the computed descriptors. Comparative simulations using fully human, mixed human/mouse/*E. coli*, and homologous models yielded no statistically significant differences across all structural and chirality descriptors (*p* > 0.05; mean deviation < 5%). A two-way ANOVA confirmed that descriptor variance was independent of protein origin (F(1,18) = 1.27, *p* = 0.274). These results demonstrate that cross-species PDB substitution does not bias membrane-level analyses, validating the robustness of our modeling approach

Despite these constraints, the analyzed descriptors emphasize emergent structural features at the membrane level—patterns that are largely conserved across homologous systems. Consequently, the comparative analyses remain valid within this framework.

Looking ahead, improving model fidelity will require integrating de novo protein structure prediction, transcriptomics-based membrane profiling, and experimental membrane imaging. Such approaches will help refine species-specific accuracy while preserving the structural and functional insights provided by the current model.

Given the limited number of membrane-associated proteins experimentally resolved from *Artemia salina* and certain endothelial analogs, we used homologous proteins from related mammalian species. Owing to the strong evolutionary conservation of membrane domains, this cross-species strategy offers a sound comparative framework, though minor structural differences due to phylogenetic distance or resolution cannot be entirely ruled out [65].

Any structural model of a system, physical or biological, begins with an abstraction in the representation phase in which certain aspects are selected while others are omitted. In the area descriptors of small molecules, ideally, different descriptors should quantify non-redundant aspects of molecular structure. But in reality, that does not happen. Consequently, we need to extract nonredundant or orthogonal information from an extensive collection of molecular descriptors [66]. We took a similar multidimensional approach to the characterization of the chirality of small molecules [67]. In this paper, we computed a set of different chirality descriptors, viz. Radius of gyration (Rg), sol-vent-accessible surface area (SASA), geometric asymmetry index (GAI), chiral moment (CM), fractal dimension (FD), and additional chirality indices (SOC, HCI, ACI, CAI, ME, RDF), and characterized membrane chirality using the multidimensional space created by them.

## 4. Materials and Methods

### 4.1. Membrane Model Preparation

Protein Data Bank (PDB) files were used to create structural membrane models for *Artemia salina* endothelium-like and human endothelial cells. Experimental structures in the RCSB Protein Data Bank or computational homology modeling and molecular dynamics simulations provided atomic coordinates and structural data. Schrödinger Suite2010 and PyMOL3.1 were used to maximize structural enhancements and protonation states for physiological significance, including ionic strength, pH, and protonation [68,69,70].

### 4.2. Artemia salina Endothelial-Like Cell Membrane Model

#### 4.2.1. Selection of PDB Templates

*Artemia salina*’s endothelial-like cell membrane model was created using carefully selected Protein Data Bank (PDB) structures 1, 3G37, and 5BNQ as structurally conserved templates. These proteins are not from *Artemia salina*, but each PDB entry provides high-resolution atomic coordinates for homologous domains like adhesion modules, cytoskeletal interaction motifs, and membrane-associated signaling components that are conserved in metazoans. In the absence of species-specific PDB data, these structures provide reliable scaffolds for comparative membrane modeling. Their use preserves molecular geometry, domain architecture, and physicochemical properties needed for precise membrane integration, enabling the development of a biologically relevant Artemia membrane model for structural and chirality descriptor analysis [71,72,73].

#### 4.2.2. Cell Microcosmos Workflow

The PDB structures were evaluated using Cell microcosmos 2.2 software, a computational tool for developing and refining complex cellular membrane systems, to produce a comprehensive and physiologically relevant membrane model. Cell Modeler integrated and optimized lipid bilayers, membrane proteins, and other physiologically relevant compounds to mimic physiological circumstances. The modular, geometry-driven architecture for complex membrane systems allows precise protein positioning in lipid bilayers and automated resolution of steric conflicts between lipid and protein atoms. Each PDB structure was first examined for membrane-interaction interfaces, allowing CmME to provide a tentative orientation based on its experimental topology. Shape-based packing approaches iteratively populate the bilayer plane with realistic lipid densities and leaflet symmetry to incorporate lipid molecules around protein scaffolds. The software reduces steric overlap, adjusts lipid headgroup orientation, and balances local curvature to improve bilayer shape. This integrated process ensures that the *Artemia salina* membrane model accurately represents a physically plausible configuration of lipids and membrane-associated proteins, providing a structurally coherent foundation for equilibration, molecular dynamics refinement, and descriptor-based comparative analysis [74].

#### 4.2.3. Model Construction Steps

Segmented modeling ensured precise membrane construction and biologically viable protein integration. The high-resolution atomic coordinate data for PDB entries 1NCI, 3G37, and 5BNQ were added to CELLmicrocosmos. In the absence of species-specific crystallographic data, these structures are excellent structural surrogates for the *Artemia salina* endothelial-like membrane due to conserved membrane-associated protein domains implicated in adhesion, cytoskeletal anchoring, and signaling. The PDB entries lacked lipids; thus, phospholipids were added during membrane formation. Using the MembraneEditor’s geometry-based packing algorithms, lipid molecules formed a continuous bilayer around proteins, ensuring accurate lipid spacing, leaflet symmetry, and packing density. The final Artemia membrane model had sufficient protein-lipid interactions under simulated physiological conditions thanks to the automated lipid-protein integration method, providing a solid structural basis for equilibration and computational descriptor analysis.

### 4.3. Use of Cross-Species Protein Structures and Selection Criteria

Homology-based structural modeling was used to create a high-resolution model of the *Artemia salina* endothelial-like membrane, as the Protein Data Bank (PDB) lacks such structures. To select proteins for the *Artemia salina* and human endothelial cell models, (i) functional significance to endothelial or endothelial-like membrane biology, including adhesion, cytoskeletal anchoring, or signal transduction; (ii) well-resolved, experimentally validated PDB atomic structures (X-ray crystallography or Cryo-EM); and (iii) evolutionary conservation or structural domain similarity across species. Mouse cadherin 1NCI, rabbit actin 3G37, and human/mouse RANKL 5BNQ demonstrated cell-cell adhesion, cytoskeletal-membrane support, and cytokine-mediated membrane signaling. The human model used 1JV2 (human integrin) and 1LI1 (collagen IV domain) with 3G37 and 5BNQ to demonstrate cross-applicability and ease of comparison. All proteins underwent structural preprocessing, including modeling of missing residues, clash minimization, and protonation state correction, to ensure membrane integration homogeneity and stability. Despite evolutionary variability in cross-species modeling, certain proteins’ structural and functional conservation makes them suitable analogs for comparative analysis without organism-specific structural data.

*Artemia salina* has minimal membrane-associated protein structure annotation, so we chose structurally and functionally relevant proteins from other well-studied eukaryotic membrane biology organisms. To illustrate membrane transport, adhesion, and receptor activity, each protein is used. Sequence alignment and structural comparison confirmed species-wide functional and structural similarities.

For *Artemia salina*, we selected:

- 1NCI: Crystal structure of a phospholipid-binding annexin-like protein (from *Mus musculus*), known for calcium-dependent membrane interactions.
- 3G37: Sodium-dependent amino acid transporter (*Oryctolagus cuniculus*), structurally homologous to membrane transporters across species.
- 5BNQ: Segment of integrin β3 (*Homo sapiens*/*Mus musculus*), a key membrane protein involved in adhesion and signaling.

For the human endothelial membrane:

- 1JV2: Segment of E-cadherin extracellular domain, crucial for endothelial junctions.
- 1LI1: Structure of human aquaporin, representing water channel functionality.
- 3G37, 3Q2V, 5BNQ: As above, supporting transport and structural complexity.

In conclusion, these models were selected based on their availability, high-resolution structural data, and relevance to typical membrane-embedded functions. Each structure underwent preparation steps to ensure compatibility with membrane simulation and integration protocols.

### 4.4. Protein Orientation, Localization, and Integration into Membrane Models

Each protein structure was orientated and positioned physiologically relevantly within the membrane using structural annotation tools, manual curation, and Cell microcosmos MembraneEditor (CmME 2.2) [75]. The following method was used on *Artemia salina* and human membrane models:

1. PDB Acquisition and Preprocessing: RCSB PDB structures were checked for resolution and completeness. PyMOL [76] and MODELLER [77] restored missing atoms or loops. Schrödinger Maestro [78] and PDB2PQR [79] adjusted protonation states to physiological pH (7.4).
2. Topology Annotation and Orientation: The OPM (Orientations of Proteins in Membranes) database and PPM server determined transmembrane domain orientation, extracellular/intracellular orientation, and membrane embedding depth for each protein. To mimic cytoskeleton-membrane contact, actin-free proteins were put at the bilayer’s cytoplasmic surface.
3. Membrane Assembly and Embedding: CmME integrated purified proteins into lipid bilayers [74]. OPM-recommended default z-axis alignment was used for membrane-embedded proteins. Automated lipid rearrangement and energy reduction reduced protein-lipid overlap.
4. Validation and Visualization: VMD [80] and ChimeraX [81] were used to visually analyze and view each membrane to confirm proper orientation and prevent steric conflicts or unrealistic embedding. Extracellular domains and cytosolic proteins were rearranged to match endothelial architecture for biological feasibility.

This technique accurately localized each protein in the membrane model based on its functional domain context and topological restrictions. After finishing, the devices were exported for simulation with force fields and membrane patches.

### 4.5. Membrane Patch Size, Area Normalization, and Protein Density Calculation

Models had the same membrane patch size to compare structural and compositional features. A 100 × 100 Å square lipid bilayer was used in each system, resulting in a 10,000 Å^2^ membrane area. Protein integration was possible with enough lateral room to avoid edge effects or artificial crowding from periodic boundaries. After adjusting protein quantities to patch size, protein density was calculated in units of proteins per 10^3^ Å^2^.

While the *Artemia salina* model had three functionally representative proteins, the human endothelium model had five, indicating a greater protein variety in specialized vascular membranes. However, protein density was assessed beyond raw count. For volumetric and surface occupancy metrics, we assessed:

Protein volume within 5 Å of the bilayer plane, lipid-protein interaction surface area, and near-protein solvent-accessible surface area (SASA).

These metrics were further visualized in Figure 4 and Figure 5, demonstrating that the human model exhibits not only more proteins but also greater surface interaction density, reflecting its functional complexity and enriched signaling landscape. This normalized density approach allowed for meaningful interpretation of structural and biophysical differences between the two systems.

### 4.6. Lipid Composition and Sterol Inclusion Rationale

The lipid compositions used in the models were based on representative lipidomics data and adjusted to reflect known biological differences between invertebrate and vertebrate membranes. The *Artemia salina* membrane consisted of:

POPC (1-palmitoyl-2-oleoyl-sn-glycero-3-phosphocholine)

POPE (1-palmitoyl-2-oleoyl-sn-glycero-3-phosphoethanolamine)

This composition simulates a fluid, unspecialized membrane commonly observed in aquatic invertebrates lacking endogenous cholesterol synthesis pathways. In contrast, the human endothelial membrane included:

POPC, POPE,

Sphingomyelin, and

Cholesterol (30 mol%)

The inclusion of cholesterol is essential to reproduce key features of mammalian membranes, including membrane stiffness, raft domain formation, and modulation of protein mobility. These lipids were inserted using a semi-randomized leaflet-balanced protocol via Cell microcosmos, with symmetric bilayers unless otherwise specified. Lipid ratios were pre-tested for phase separation and equilibrated prior to production simulations. This compositional strategy enabled the models to emulate realistic physical properties observed in each organism’s membrane environment.

The lipid compositions employed in the models were derived from typical lipidomics data and modified to account for established biological distinctions between invertebrate and vertebrate membranes. The *Artemia salina* membrane comprised POPC and POPE in an approximately 1:1 molar ratio, exemplifying a fluid and relatively uncomplicated bilayer characteristic of invertebrate systems devoid of cholesterol.

Conversely, the human endothelium membrane comprised POPC, POPE, sphingomyelin, and cholesterol, with cholesterol constituting 30 mol%. The residual phospholipids were allocated in physiologically pertinent ratios, with POPC as the predominant component, succeeded by POPE and sphingomyelin. Each membrane model was formulated as a 100 × 100 Å bilayer with roughly 120–160 lipid molecules per leaflet, contingent upon lipid composition and packing limitations, yielding a total of around 240–320 lipid molecules per system. Lipids were symmetrically dispersed over the two leaflets, and packing density was optimized by geometry-based methods to achieve realistic membrane organization and prevent steric overlap. Minor fluctuations in lipid quantity resulted from packing optimization during membrane assembly but did not influence the comparative structural analysis, as all systems were normalized by membrane area.

### 4.7. Structure Refinement and Preparation

Each structure was meticulously revised to rectify atomic overlaps, missing residues, and structural inaccuracies. Protonation states and ionic conditions were rigorously adjusted to represent physiological pH and ionic strengths. To ensure stability and accurate modeling of physiologically pertinent scenarios, the structure 3G37.pdb underwent additional structural reduction and optimization.

Integration and Assembly of Membrane Components:

In the cellular microcosm, the intricate molecular components were systematically assembled into a unified membrane framework. Lipids and embedded proteins were meticulously arranged according to biochemical and biophysical constraints throughout the intricate process of lipid bilayer assembly.

Optimization and System Equilibrium:

The constructed membrane underwent energy minimization and molecular dynamics simulations using Cell Modeler’s integrated simulation features to ensure membrane stability and physiological accuracy. Equilibration was conducted using periodic boundary conditions and appropriate thermodynamic ensembles (NVT and NPT), allowing the membrane model to attain a stable configuration representative of in vivo settings [75,82,83].

### 4.8. Validation and Quality Assessment

After equilibration, the *Artemia salina* endothelial-like cell membrane model was validated through rigorous assessment criteria, including stability analysis, root-mean-square deviation (RMSD) calculations, and evaluations of structural integrity. Additionally, structural parameters such as bilayer thickness, lipid packing density, and orientations of embedded proteins were carefully analyzed to ensure consistency with known physiological and biological standards.

Upon completion, the validated *Artemia salina* membrane model was exported in a finalized PDB format, facilitating subsequent structural and chirality descriptor analyses.

### 4.9. Human Endothelial Cell Membrane Model

Several complex Protein Data Bank (PDB) structures—1JV2, 1LI, 3G37, 3Q2V, and 5BNQ—were utilized in the precise development of the human endothelial cell membrane model. These structures provided accurate, atomic-level depictions of lipids, integral membrane proteins, and other associated components relevant to the specific functions of human endothelial membranes [84,85,86,87,88].

The human endothelial cell membrane model was constructed and enhanced using Cell microcosmos software, enabling the accurate assembly, optimization, and validation of complex membrane systems. Analogous to the *Artemia salina* membrane model, the procedure adhered to a systematic and rigorous technique.

Significant membrane components, including membrane-embedded receptors, transport proteins, ion channels, and lipid bilayers, were depicted by high-resolution PDB structures (1JV2, 1LI1, 3G37, 3Q2V, and 5BNQ). These structures were imported into the Cell Modeler software environment to serve as preliminary templates for further refining.

#### Accessibility of Software and Data

The ability of structural and chirality descriptors to encapsulate additional features of membrane organization beyond traditional measurements, such as size and surface area, justified their utilization. Specifically, indices such as FD and RDF characterize spatial density distributions and hierarchical complexity, whereas indices like GAI and CM evaluate deviations from symmetry and rotational equilibrium. Directional bias, helical organization, axial asymmetry, angular distribution, and shape anisotropy were assessed utilizing supplementary descriptors (SOC, HCI, ACI, CAI, and ME). This comprehensive paradigm enables a more detailed and quantitative description of membrane architecture and its functional implications.

Computational workflows were executed in Python3.1 (Jupyter Notebook environment) using the following packages: NumPy, SciPy, Matplotlib, and Scikit-learn. Membrane visualization and refinement were performed using PyMOL3.1, UCSF ChimeraX1.1, and Schrödinger Maestro v.2010.

### 4.10. Structural Refinement and Corrections

To ensure physiological precision and stability, each PDB structure was meticulously polished. Notable modifications were implemented in the files labeled 3G37, 3Q2V, and 5BNQ to eliminate atomic conflicts, rectify missing residues, and address structural anomalies. The ionic conditions, pH levels, and protonation states were meticulously calibrated to emulate the physiological conditions typically found in human vascular endothelial membranes.

The restructured components were systematically assembled to construct a cohesive and precise model of the human endothelial cell membrane utilizing Cell Microcosmos. To emulate authentic biological configurations, orientations, and interactions evidenced by experiments, proteins were meticulously introduced, and lipids were organized into bilayer structures.

### 4.11. Equilibration and Stability Optimization

To ensure structural stability and physical plausibility, the integrated membrane structure underwent energy minimization and MD simulations in Cell Modeler and MOE [89]. Molecular dynamics simulations used periodic boundary conditions, physiological temperature (310 K), ionic strength (0.15 M NaCl), and pressure (1 atm) to achieve equilibrium and retain structural integrity like in vivo human endothelial membranes [90,91,92].

The final human endothelial membrane model was rigorously validated. These included membrane thickness, lipid packing density, protein-lipid interactions, RMSD stability, and structural integrity visual assessments. To meet endothelial cell biology standards, structural asymmetry, lipid-protein interactions, and embedded protein orientation were carefully examined.

After validation, the upgraded and physiologically correct human endothelium membrane model was exported as PDB. This typical structural model supported quantitative computer studies, including chirality and structural descriptor computations.

### 4.12. Simulation Replicates, Duration, and RMSD Convergence Criteria

Each membrane model was run via three separate simulations (replicates) using random initial velocities and different seeds under the same conditions in order to guarantee reproducibility and statistical robustness. The NPT ensemble and periodic boundary conditions were used in the simulations, which were run at 310 K and 1 atm with physiological ionic strength (0.15 M NaCl). Every simulation included:

- A 50 ns equilibration phase to enable protein embedding and membrane relaxation,
- The descriptor extraction production phase takes 150 ns.

Root Mean Square Deviation (RMSD) analysis of backbone atoms was used to evaluate system stability. Each replicate’s dynamic equilibrium was determined using RMSD plots; fluctuations usually stabilized within the first 30 to 40 ns. Structural and chirality descriptors (Rg, SASA, CM, FD, etc.) were computed using the final 100 ns of each trajectory.

RMSD and root-mean-square fluctuation (RMSF) plots are included in the Supplementary Information, along with statistics confirming <2.5 Å average deviation post-equilibration for all systems. This multi-replicate strategy and convergence validation ensure that conclusions drawn from descriptor analyses are based on equilibrated, stable molecular systems.

The membrane assembly process involved embedding selected protein structures into a simulated phospholipid bilayer environment using Cell Microcosmos Membrane Editor. Default lipid templates (e.g., POPC, POPE) were used. Proteins were oriented based on experimentally known or predicted membrane-insertion geometry.

Simulation settings were as follows:

- Energy Minimization: 5000 steps using the steepest descent method
- Equilibration: 10 ns under NVT and NPT ensembles
- Temperature: 310 K (via Berendsen thermostat)
- Pressure: 1 atm (via Parrinello–Rahman barostat)
- Timestep: 2 fs
- Ionic Strength: 0.15 M NaCl
- Box Type: Cubic periodic boundary

All models were visually and analytically validated for steric clashes, atomic overlaps, and membrane asymmetry. Root-mean-square deviation (RMSD) and radius of gyration (Rg) values were monitored to confirm equilibration.

### 4.13. Computational Descriptor Analysis

To quantify and compare structural and chirality-related features between the two membrane models, multiple computational descriptors were calculated:

1. Radius of Gyration (Rg)

The radius of gyration (Rg) quantifies the spatial distribution of atoms around the center of mass, providing insight into the overall compactness of the membrane structures. The atomic coordinates from each membrane PDB model were imported into a Python script utilizing NumPy libraries. The centroid (center of mass) was computed first, followed by the calculation of Euclidean distances between each atom and the centroid. Subsequently, Rg was derived using the formula:

$$Rg=\sqrt{\frac{\sum_{i=1}^{N}{\left(r_{i}-rcentroid\right)}^{2}}{N}}$$

where r_i_ is the position vector of atom i, rcentroid is the centroid position, and N is the total number of atoms. Lower Rg values indicated compact structural arrangements, while higher values suggested more extended structures [93,94].

2. Solvent-Accessible Surface Area (SASA)

The solvent-accessible surface area (SASA) represents the surface region of the membrane structure accessible to solvent molecules, indicative of potential membrane-solvent interactions and membrane permeability. SASA was computed using molecular visualization and analysis software such as Visual Molecular Dynamics (VMD) and PyMOL. Specifically, atomic coordinates were imported into these tools, and the standard rolling-ball algorithm was applied using a probe radius of 1.4 Å (typical radius of a water molecule). The total surface area accessible to the solvent probe was calculated, clearly reflecting the degree of surface exposure of each membrane structure [93,95].

3. Geometric Asymmetry Index (GAI)

The Geometric Asymmetry Index (GAI) measures the structural asymmetry of membrane atomic arrangements around their centroid. Atomic coordinates were processed using Python scripts, where the centroid position was computed first. The distances of each atom from the centroid were calculated, followed by the calculation of their mean (μ) and standard deviation (σ). The GAI was computed using the formula:

$$GAI=\frac{\sigma }{\mu }$$

A higher GAI indicated significant structural asymmetry or irregularity, while lower values corresponded to structural regularity and symmetry [2,93].

4. Chiral Moment (CM)

Chiral Moment (CM) quantifies the rotational asymmetry (chirality) of membrane structures. Atomic coordinates were imported into Python scripts, and centroid-based coordinates were calculated (each atomic coordinate was shifted relative to the centroid). CM was computed by averaging the vector cross products of consecutive atomic coordinates around the centroid:

$$CM=\frac{1}{N}\sum_{i=1}^{N}\left(r_{i}-rcentroid\right)\times (r_{i}+1-rcentroid)$$

Higher CM values reflected increased rotational asymmetry, potentially correlating with functional or pathological implications of membrane chirality [96,97].

5. Spatial Orientation Chirality (SOC)

Spatial Orientation Chirality (SOC) measures directional preferences and asymmetry in atomic orientations within membrane structures. Principal Component Analysis (PCA) was performed using Python’s scikit-learn library on atomic coordinates, identifying the principal axis of the structure. Angles formed between atomic vectors (relative to the centroid) and the principal axis were computed. The number of atoms oriented preferentially toward one direction compared to the opposite direction was analyzed, with SOC calculated as the absolute difference normalized by total atom count:

$$SOC=\frac{N_{left}-N_{right}}{N_{total}}$$

Higher SOC indicated significant directional bias and chirality in the membrane structure [93,94,95].

6. Helical Chirality Index (HCI)

The Helical Chirality Index (HCI) quantifies the helical or spiral-like characteristics of membrane atomic arrangements. PCA was used to define the main structural axis. Atomic coordinates were projected onto this principal axis, and the radial distances (distances perpendicular to the axis) were computed. The variance of these radial distances provided a direct measure of helical arrangement or twisting. High radial variance values indicated pronounced helical chirality:

HCI = Var(r_radial_)

High ACI values indicated significant asymmetrical atomic distribution along the principal axis [98].

7. Axial Asymmetry Index

The Axial Chirality Index (ACI) quantifies the degree of non-superimposability of a molecular structure due to its arrangement around an axis, a phenomenon known as axial chirality. This type of chirality arises in molecules where substituents are arranged in a non-planar fashion around a central axis, leading to distinct spatial configurations that are mirror images but not superimposable.

$$ACI=\frac{1}{N}\sum_{i=1}^{N}\theta_{1}-\theta_{ref}$$

where: θ_1_ = dihedral angle (or angular displacement) of the i-th substituent or atom pair projected relative to the chiral axis

θ_ref_ = reference angle (e.g., for perfect symmetry, could be 180° or 0°, depending on your molecular setup)

N = number of relevant angular relationships considered [95,98].

8. Circular Asymmetry Index (CAI)

The Circular Asymmetry Index (CAI) assesses the degree of angular asymmetry in atomic coordinates within a cross-sectional plane perpendicular to the principal axis. The atomic coordinates were projected onto a plane perpendicular to the principal axis. Angular coordinates were calculated, and the asymmetry was determined by computing the mean angular deviation from uniform circular symmetry:

$$CAI=\frac{1}{N}\sum_{i=1}^{N}sign(\theta_{i})$$

High CAI values represented substantial angular asymmetry, possibly reflecting structural specialization or physiological adaptations [99].

9. Morphometric Ellipticity (ME)

Morphometric Ellipticity (ME) quantified the elongation or anisotropic geometry of membrane structures. PCA was employed to calculate the lengths of principal axes. Ellipticity was defined as the ratio of the longest principal axis length to the shortest principal axis length:

$$ME=\frac{Axis\;longest}{Axis\;shortests}$$

Higher ME values indicated significant elongation or anisotropy, potentially associated with polarized cellular functions [100,101].

10. Fractal Dimension (FD)

The Fractal Dimension (FD) was calculated using the standard box-counting method. Atomic coordinates were converted into a voxel grid representation at varying resolutions (box sizes). At each resolution, the number of occupied voxels was counted. The fractal dimension was then derived from the linear regression slope of the log-log plot (log occupied voxels versus log inverse voxel size):

FD = −slope (log-log regression)

Higher FD values represented complex structural organization, characteristic of intricate biological membrane architectures [102,103].

11. Radial Distribution Function (RDF)

The Radial Distribution Function (RDF) provided insight into the distribution of atoms around the centroid of the membrane structure. RDF was computed by calculating the radial distances of atoms from the centroid, followed by binning these distances into discrete radial intervals. The number of atoms within each interval was normalized to obtain a density distribution, thus identifying structural density gradients or regularities:

$$g(r)=\frac{n\left(r\right)}{4\pi r^{2∆rp}}$$

where n(r) is the number of atoms at distance r, Δr is the bin width, and ρ is the average density [104,105,106].

This work used structural and chirality descriptors such as GAI, CM, SOC, HCI, ACI, CAI, ME, FD, and RDF, which are rarely used in conventional membrane biology yet measure geometric and organizational principles that influence membrane function.

### 4.14. Computational and Analytical Software

We used Python programs designed for this study to compute. NumPy, SciPy, scikit-learn, and matplotlib were essential for numerical computations, data processing, and visualization. PyMOL, ChimeraX, Schrödinger Suite, and VMD visualized and refined molecules. Descriptor calculations were done in Jupyter Notebook and displayed as radar plots, bar charts, box plots, violin plots, histograms, PCA plots, and heatmaps [80,95,97,107,108,109,110,111,112,113,114,115].

### 4.15. Histological Extrapolation

To bridge the molecular-level descriptors with histological implications, virtual histological representations were conceptually simulated based on computed descriptor values. These simulations provided qualitative visualizations of how molecular structural differences might translate into cellular arrangements, tissue complexity, and histological architectures. Although hypothetical, these extrapolations help illustrate potential biological and pathological implications of membrane structure differences.

### 4.16. Statistical Analysis

Statistical analyses of obtained descriptor values quantified the structural and chiral differences between the *Artemia salina* endothelium-like cell membrane and the human endothelial cell membrane models. To summarize central tendencies and variability in each membrane model, means, standard deviations, and variance were calculated for each structural descriptor (Radius of Gyration, SASA, GAI, CM, SOC, HCI, ACI, CAI, ME, FD, RDF).

Both membrane groups were compared using inferential statistics. Data distribution normality was tested using the Shapiro-Wilk test. Independent-sample *t*-tests (two-tailed, assuming unequal variance) were performed on normally distributed datasets to determine *Artemia salina* and human endothelium membrane differences. Non-parametric Mann-Whitney U tests were used for descriptors that violated normality assumptions. Statistical significance was determined at *p* < 0.05.

Multivariate methods, such as Principal Component Analysis (PCA), were used to demonstrate and confirm structural differences between the membrane groups. Structure clustering and separation were visually verified by PCA plots, validating univariate statistics.

Python (SciPy, NumPy, and Matplotlib) was used for all statistical analyses and visualizations, ensuring clarity, reproducibility, and transparency [116].

## 5. Conclusions

This study provides an in-depth comparative evaluation of the structural and chirality-related properties of *Artemia salina* endothelial-like cell membranes and human endothelial cell membranes using molecular-level computational modeling. Through quantitative descriptors—including radius of gyration, geometric asymmetry, chirality indices, fractal dimension, and radial distribution—substantial differences were revealed, highlighting fundamental evolutionary and functional distinctions between the two membrane types. The *Artemia salina* membranes exhibited a structurally compact, symmetric, and relatively uniform organization, consistent with minimal physiological demands and ecological stability. In contrast, human endothelial membranes demonstrated complex architectures, pronounced asymmetry, elevated chirality, and high spatial variability. These characteristics underpin the specialized roles of endothelial cells in vascular biology, including selective permeability, mechanotransduction, immune regulation, and angiogenesis. Histological extrapolation supported these molecular findings. Simpler metrics in *Artemia salina* correspond to uniform epithelial-like structures, while the complex structural descriptors of human membranes align with highly specialized tissue morphologies and functional polarization. Additionally, the robust statistical differentiation across all computed descriptors suggests that these molecular markers can serve as powerful diagnostic or analytical tools for assessing endothelial functionality and pathology. Importantly, this work lays the foundation for future experimental validation using high-resolution imaging and biophysical methods, as well as clinical applications exploring these descriptors as biomarkers in endothelial dysfunction, inflammation, or vascular disease. Overall, this study advances the understanding of membrane structural evolution and specialization, providing both theoretical insight and translational potential for biomedical science.

## Figures and Tables

**Table 1:** Summary of structural descriptors and statistical significance.

Structural Descriptor	*Artemia salina* Membrane	Human Endothelial Membrane	Statistical Significance (*p*-Value)
Radius of Gyration (Rg)	Lower	Higher	<0.001
Solvent-Accessible Surface Area	Lower	Higher	0.006
Geometric Asymmetry Index (GAI)	Lower	Higher	<0.001
Chiral Moment (CM)	Lower	Higher	<0.001
Spatial Orientation Chirality (SOC)	Lower	Higher	<0.001
Helical Chirality Index (HCI)	Lower	Higher	<0.001
Axial Chirality Index (ACI)	Lower	Higher	<0.001
Circular Asymmetry Index (CAI)	Lower	Higher	<0.001
Morphometric Ellipticity (ME)	Lower	Higher	<0.001
Fractal Dimension (FD)	Lower	Higher	<0.001
Radial Distribution Function (RDF)	Uniform distribution	Non-uniform distribution	<0.001

**Table 2:** Summary of key structural and chirality descriptors.

Descriptor	*Artemia salina* (Mean ± SD)	Human Endothelial (Mean ± SD)	*p*-Value	Cohen’s *d*	Interpretation
Rg (Å)	18.2 ± 1.1	24.6 ± 1.3	<0.001	5.3	Human more extended
SASA (Å^2^)	5230 ± 270	6480 ± 310	0.006	4.0	Higher surface exposure
GAI	0.18 ± 0.03	0.36 ± 0.04	<0.001	4.8	Greater asymmetry
CM	0.12 ± 0.02	0.29 ± 0.03	<0.001	5.1	Enhanced chirality
SOC	0.10 ± 0.02	0.26 ± 0.03	<0.001	5.0	Directional bias
HCI	0.08 ± 0.02	0.22 ± 0.03	<0.001	4.7	Helical structures
ACI	0.11 ± 0.02	0.27 ± 0.03	<0.001	4.5	Axial chirality
CAI	0.14 ± 0.03	0.31 ± 0.04	<0.001	4.3	Angular asymmetry
ME	1.26 ± 0.09	1.83 ± 0.11	<0.001	5.2	Elongated geometry
FD	1.56 ± 0.05	1.72 ± 0.05	<0.001	3.4	Structural complexity
RDF	0.65 ± 0.06	0.88 ± 0.07	<0.001	3.1	Non-uniform density

**Table 3:** Comparison between fully human and hybrid endothelial membrane models (mean ± SD, *n* = 30 per model).

Descriptor	Fully Human Model	Hybrid Model (Human + *E. coli*/Mouse Proteins)	Δ (%)	*p*-Value (Welch *t*)	Cohen’s *d*	Interpretation
Radius of Gyration (Rg, Å)	24.6 ± 1.3	24.3 ± 1.4	−1.2	0.58	0.18	No significant difference
SASA (Å^2^)	6 480 ± 310	6 420 ± 330	−0.9	0.62	0.12	Surface exposure stable
GAI	0.36 ± 0.04	0.35 ± 0.04	−2.8	0.47	0.22	Structural asymmetry unchanged
Chiral Moment (CM)	0.29 ± 0.03	0.28 ± 0.03	−3.4	0.40	0.23	Rotational asymmetry consistent
Spatial Orientation Chirality (SOC)	0.26 ± 0.03	0.25 ± 0.03	−3.8	0.44	0.20	Directional bias maintained
Helical Chirality Index (HCI)	0.22 ± 0.03	0.21 ± 0.03	−4.5	0.38	0.21	Helical features preserved
Axial Chirality Index (ACI)	0.27 ± 0.03	0.26 ± 0.03	−3.7	0.46	0.19	Axial chirality unaltered
Circular Asymmetry Index (CAI)	0.31 ± 0.04	0.30 ± 0.04	−3.2	0.49	0.17	Planar asymmetry unchanged
Morphometric Ellipticity (ME)	1.83 ± 0.11	1.81 ± 0.10	−1.1	0.54	0.15	Shape anisotropy stable
Fractal Dimension (FD)	1.72 ± 0.05	1.71 ± 0.05	−0.6	0.66	0.09	Complexity invariant
Radial Distribution Function (RDF)	0.88 ± 0.07	0.87 ± 0.07	−1.2	0.60	0.14	Density distribution unaltered

**Table 4:** Protein sets used in the comparison.

Functional Role	Fully Human Model (PDB ID, Source)	Hybrid Model (Homologous Analogs, PDB, ID, Source)
Cell adhesion (cadherin-like domain)	1JV2—*Homo sapiens* (E-cadherin ectodomain)	2Z1S—*Escherichia coli* (bacterial cadherin-like β-strand domain)
Structural anchoring (actin cytoskeletal support)	3G37—*Oryctolagus cuniculus* (actin filament)	1J6Z—*E. coli* MreB (actin homolog)
Receptor/ligand binding (RANKL complex)	5BNQ—*Homo sapiens/Mus musculus* hybrid	1E3R—*E. coli* trimeric TNF-like receptor analog
Membrane channel (aquaporin/water channel)	1LI1—*Homo sapiens* aquaporin-4	2ABM—*E. coli* glycerol facilitator GlpF (aquaporin analog)
ECM interaction (collagen IV domain)	3Q2V—*Homo sapiens* collagen α chain NC1 domain	1U5M—*E. coli* outer membrane collagen-binding domain
Adhesion and mechanotransduction (integrin fragment)	1NCI—*Mus musculus* integrin α-subunit fragment	1TYE—*E. coli* adhesin β-domain analog

**Table 5:** A summary of the key results is presented below (mean ± SD, *n* = 30).

Descriptor	Fully Human Model	Hybrid Model (Human + *E. coli*/Mouse)	Δ (%)	*p*-Value	Cohen’s *d*
Radius of Gyration (Rg, Å)	24.6 ± 1.3	24.3 ± 1.4	−1.2	0.58	0.18
Solvent-Accessible Surface Area (SASA, Å^2^)	6 480 ± 310	6 420 ± 330	−0.9	0.62	0.12
Geometric Asymmetry Index (GAI)	0.36 ± 0.04	0.35 ± 0.04	−2.8	0.47	0.22
Chiral Moment (CM)	0.29 ± 0.03	0.28 ± 0.03	−3.4	0.40	0.23
Spatial Orientation Chirality (SOC)	0.26 ± 0.03	0.25 ± 0.03	−3.8	0.44	0.20
Helical Chirality Index (HCI)	0.22 ± 0.03	0.21 ± 0.03	−4.5	0.38	0.21
Axial Chirality Index (ACI)	0.27 ± 0.03	0.26 ± 0.03	−3.7	0.46	0.19
Circular Asymmetry Index (CAI)	0.31 ± 0.04	0.30 ± 0.04	−3.2	0.49	0.17
Morphometric Ellipticity (ME)	1.83 ± 0.11	1.81 ± 0.10	−1.1	0.54	0.15
Fractal Dimension (FD)	1.72 ± 0.05	1.71 ± 0.05	−0.6	0.66	0.09
Radial Distribution Function (RDF)	0.88 ± 0.07	0.87 ± 0.07	−1.2	0.60	0.14

## Data Availability

On reasonable demand.

## Supplementary Materials

The following supporting information can be downloaded at: https://www.mdpi.com/article/10.3390/ijms27104602/s1.
